# Co-administration of exercise training and melatonin on the function of diabetic heart tissue: a systematic review and meta-analysis of rodent models

**DOI:** 10.1186/s13098-023-01045-6

**Published:** 2023-04-01

**Authors:** Afshin Rahbarghazi, Karim Azali Alamdari, Reza Rahbarghazi, Hanieh Salehi-Pourmehr

**Affiliations:** 1 Department of Physical Education and Sports Sciences, Faculty of Educational Science and Psychology, University of Mohaghegh Ardabil, Daneshgah Street, Ardabil, 56199-11367 Iran; 2 Stem Cell Research Center, Tabriz University of Medical Sciences, Imam Reza St., Golgasht St, Tabriz, Iran; 3 Department of Sport Sciences, Azarbaijan Shahid Madani University, Tabriz, Iran; 4 Tuberculosis and Lung Disease Research Center, Tabriz University of Medical Sciences, Tabriz, Iran; 5 Department of Applied Cell Sciences, Faculty of Advanced Medical Sciences, Tabriz University of Medical Sciences, Tabriz, Iran; 6 Research Center for Evidence-Based Medicine, Iranian EBM Centre: A Joanna Briggs Institute (JBI) Center of Excellence, Tabriz University of Medical Sciences, Tabriz, Iran

**Keywords:** Diabetes mellitus, Melatonin, Exercise, Cardiac tissue, Therapeutic effects

## Abstract

**Purpose:**

Diabetes mellitus (DM), a hyperglycemic condition, occurs due to the failure of insulin secretion and resistance. This study investigated the combined effects of exercise training and melatonin (Mel) on the function of heart tissue in diabetic rodent models.

**Methods:**

A systematic search was conducted in Embase, ProQuest, Cochrane library, Clinicaltrial.gov, WHO, Google Scholar, PubMed, Ovid, Scopus, Web of Science, Ongoing Trials Registers, and Conference Proceedings in July 2022 with no limit of date or language. All trials associated with the effect of Mel and exercise in diabetic rodent models were included. Of the 962 relevant publications, 58 studies met our inclusion criteria as follows; Mel and type 1 DM (16 studies), Mel and type 2 DM (6 studies), exercise and type 1 DM (24 studies), and exercise and type 2 DM (12 studies). Meta-analysis of the data was done using the Mantel Haenszel method.

**Results:**

In most of these studies, antioxidant status and oxidative stress, inflammatory response, apoptosis rate, lipid profiles, and glucose levels were monitored in diabetic heart tissue. According to our findings, both Mel and exercise can improve antioxidant capacity by activating antioxidant enzymes compared to the control diabetic groups (p < 0.05). The levels of pro-inflammatory cytokines, especially TNF-α were reduced in diabetic rodents after being treated with Mel and exercise. Apoptotic changes were diminished in diabetic rodents subjected to the Mel regime and exercise in which p53 levels and the activity of Caspases reached near normal levels (*p* < 0.05). Based on the data, both Mel and exercise can change the lipid profile in diabetic rodents, especially rats, and close it to near-to-control levels.

**Conclusion:**

These data showed that exercise and Mel can reduce the harmful effects of diabetic conditions on the heart through the regulation of lipid profile, antioxidant capacity, apoptosis, and inflammation.

## Introduction

Diabetes Mellitus (DM) is a common global health problem with different socioeconomic complications [1, 2]. According to American Diabetes Association statistics, the number of diabetic patients is expected to increase to more than 600 million people in 2035. Of note, more than 10% of the adult population suffer from DM in Iran and it is estimated that about half of this population is unaware of their diabetic conditions [3]. Predisposing factors such as lifestyle changes, obesity, genetic predisposition, urbanization, physical inactivity, and aging have led to the prevalence of DM [4]. From biochemical aspects, DM coincides with abnormal insulin secretion and insensitivity, leading to dysregulation of carbohydrate, protein, and lipid metabolism. With the progression of the diabetic condition, several pathologies such as retinopathy, nephropathy, neuropathy, and cardiovascular diseases are possible [5, 6]. Clinical studies have revealed two main types of DM. Type 1 DM (T1DM) is induced following the progressive destruction of pancreatic insulin-producing beta cells via the activity of auto-reactive T lymphocytes [7]. In contrast to T1DM, type 2 DM (T2DM) is diagnosed with abnormal insulin activity and insulin resistance (IR) in the target cells, leading to hyperglycemic conditions. About 90% of DM is associated with T2DM and a potential risk (2- to threefold) of cardiovascular diseases [8]. It has been shown that DM increases heart tissue problems by two and five times in males and females, respectively compared to non-diabetic counterparts [9]. Prolonged hyperglycemia leads to the promotion of oxidative stress which per se triggers free radical formation and lipid peroxidation. These features cause a prominent inflammatory response, apoptotic changes, and pathological conditions in cardiac tissue [2, 10, 11]. Considering the high metabolic activity in cardiomyocytes, it is logical to think that these cells are prone to injury following the production of free radicals and oxidative stress. In line with this claim, several studies have confirmed the accumulation of reactive oxygen species (ROS) in cardiac tissue under diabetic conditions [12].

Melatonin (Mel) is a lipophilic hormone produced mainly in the brain parenchyma from tryptophan [13, 14]. Regarding the existence of physical and chemical activities, Mel with specific properties can be used in the management of DM [15, 16]. The modulation of inflammation and inhibition of apoptosis is the underlying mechanisms by which Mel protects cardiomyocytes against diabetic conditions [17]. Direct diffusion and internalization via cell-membrane bound receptors help Mel to actively neutralize cellular free radicals [18, 19].

Under resting conditions, normal cardiomyocytes possess high oxidative metabolism with relatively lower antioxidant capacity. Following physical activities, intracellular levels of ROS are increased and it is thought that exercise is an important stimulus for the regulation of various antioxidants [6]. It has been indicated that regular exercise can internalize glucose and glycogen into the cytosol, and maintains the glucose at the normal range by regulating the function of insulin [20]. These features contribute to the reduction of inflammatory response in pancreatic insulin-producing cells. Data confirmed that beta cell insulin sensitivity is improved with regular exercise [21]. In the current systematic review article, we investigated the effects of exercise training and Mel on cardiac tissue function in the diabetic rodent model.

## Materials and methods

### Search strategy

A systematic search was conducted in Embase, ProQuest, Cochrane library, Clinicaltrial.gov, WHO, Google Scholar, PubMed, Ovid, Scopus, Web of Science, Ongoing Trials Registers, and Conference Proceedings in July 2022 with no limit of date or language. The list of included review articles, experiments, and contacted authors of included trials was screened for subsequent analyses. We also monitored the abstracts from the international congresses. Unpublished or incomplete experiments were scoped via researchers known to participate in similar studies.

### Inclusion and exclusion criteria

All experiments related to the application of exercise and Mel on diabetic heart tissue either mice or rats were included in this study. We excluded all studies that reported the effects of exercise or Mel on rodent non-cardiac tissues or human subjects or studies without access to the full text. The inclusion and exclusion criteria are summarized in Table 1. The title and abstract screening process was done independently by two researchers. Each author separately evaluated the full text of the selected articles. Any disagreement in different parts of the study was resolved by discussion between the reviewers until a consensus was reached.

**Table 1:** Inclusion and exclusion criteria used in this study for round-I selection

	Inclusion criteria	Exclusion criteria
Participants	Preclinical studies Studies on rodent models	In vitro studies Studies with human subjects
Intervention	Any melatonin or exercise supplementation dose heart tissue	Studies without melatonin or exercise supplementation
Comparison	Placebo or usual care; any other non-pharmacological interventions or pharmacological interventions	None
Outcomes		
Study type	Randomized clinical trials	
Language	No limit	
Year of publication	No limit	

### Data extraction

Two authors independently recorded the information using a data extraction form as follows: author, year of publication and type, animal characteristics (including strain, species, and sex) and age and weight, and diabetic disease model with details of induction protocols and Mel, characteristics of exercise training (including type, path, time, dose, and frequency of exercise), study groups, duration of intervention, study results and mechanisms (see the following sections). We collected data for the nature of the reported outcome, animal number per group, and mean ± SD or mean ± SEM. In a single publication where different experiments were shown, data were treated as independent experiments. The disagreement was resolved by consulting a third party. For data presented graphically, we monitored the values of the graphs using Universal Desktop Ruler (version 2.9) or contacted the authors of the article for details.

### Methodological quality

For this purpose, two reviewers assessed the methodological quality of the selected trials. The risk of bias was assessed through a 6-criterion appraisal checklist containing sequence generation, allocation concealment, blinding, incomplete outcome data, selective outcome reporting, and other biases. The internal validity of the enrolled studies (e.g., selection, performance, detection, and attrition bias) and other study quality measures (e.g., reporting quality, power) was assessed using a modified version of the Collaborative Approach to Meta-Analysis and Review of Animal Data from Experimental Studies (CAMARADES) quality checklist [22].

### Statistical analysis

Outcomes of interest in the current analysis were improvement of oxidative status, inflammatory and apoptotic responses, glucose levels, and positive effects on lipid profiles and myocardial damage in diabetic rodents. Meta-analysis of the data was done by using the Mantel Haenszel method with Comprehensive Meta-Analysis software (ver. 2.2; Biostat, Englewood, NJ, USA). All variables were continuous data. Mean ± SD was used to calculate the standardized mean difference and 95% confidence interval (CI). Statistical heterogeneity was analyzed using the I^2^ value and the result of the chi-square test. p < 0.05 and I^2^ > 50% were considered suggestive of statistical heterogeneity. A fixed-effect model was used when there was no statistically significant difference in the heterogeneity (P < 0.05); otherwise, a random-effect model was applied. To examine any potential publication bias in the studies, the results of the comprehensive meta-analysis are shown as Funnel plots.

## Results

### Description of studies

**Fig. 1:**
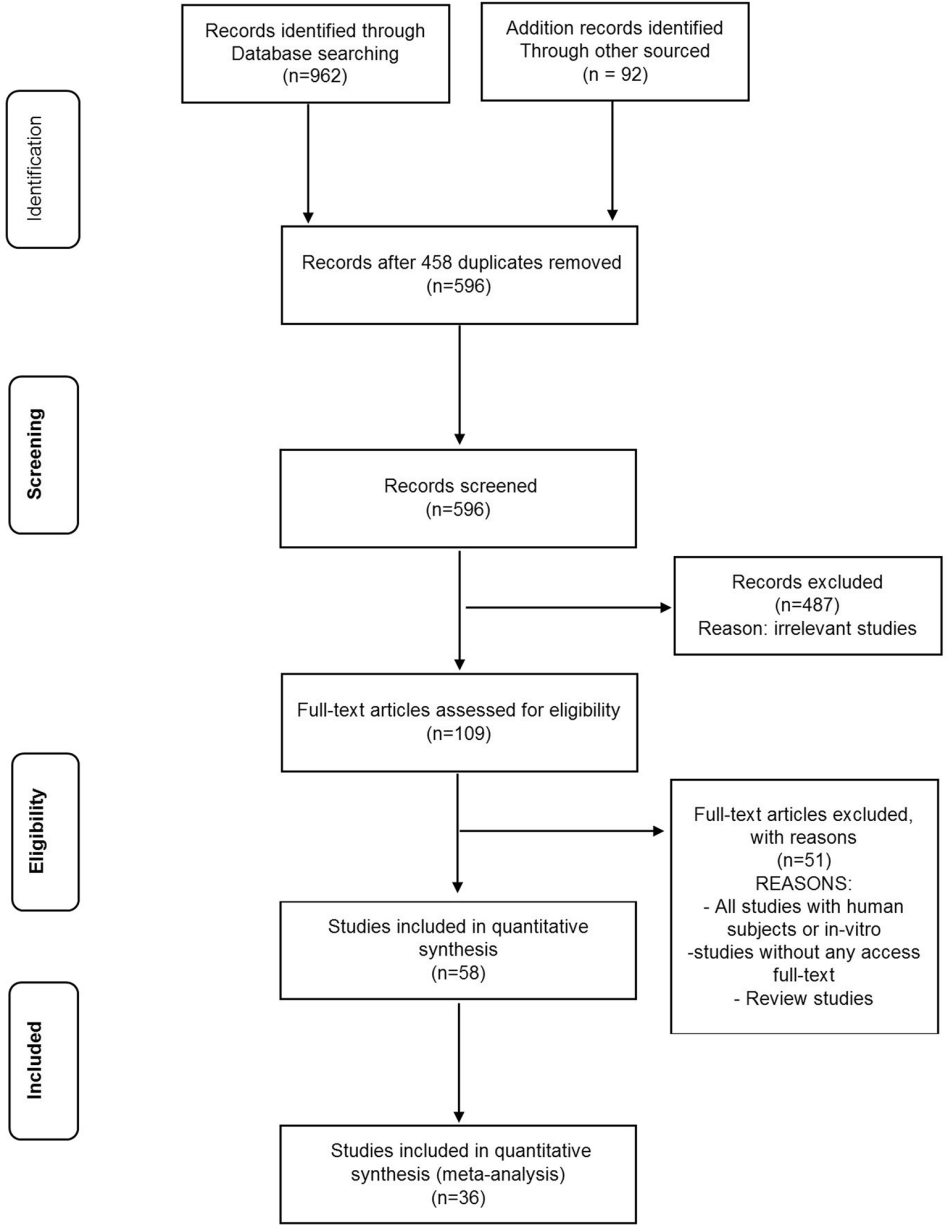
Preferred reporting items for systematic reviews and meta-analyses (PRISMA) diagram of included studies in qualitative and quantitative stages

We found 962 relevant publications during the search of electronic databases. Among them, 596 were excluded after an intensive and preliminary screening of the titles and abstracts, duplicate publications, or human subjects. The full text of 109 articles was evaluated and finally, 58 studies met our inclusion criteria related to the effects of Mel and exercise on diabetic cardiac tissue (Table 2). Among the included studies, 40 articles were conducted on T1DM, and 18 on T2DM. Mice and rats have been used in most of the publications. Heart tissue has been studied as one of the most important body tissues in diabetes. The common pathways for these changes included oxidative stress, antioxidant enzyme activity, angiogenesis, autophagy, apoptosis, and inflammatory indicators, of which 36 studies met all inclusion criteria. A flow chart for data selection is represented in Fig. 1.

**Table 2:** Characteristics of included studies in systematic review and meta-analysis

Species	Age	Weight	Type diabetic	Intervention	Sample size	The time between induction and starter intervention	Intervention duration	Outcome (s)	Mechanism	Refs.
Male Sprague Dawley rats	22 ± 2 (months)	300–325 (g)	T2DM	T2DM (a high-fat diet (62% calories obtained from fat)/ diet + 35 mg/kg of STZ by intraperitoneal + Melatonin (10 mg/kg) + Sitagliptin (20 mg/kg, i.p.) for 4 weeks	N = 42 7 groups (6 rats each) 1—control group received only thoracotomy without LAD ligation; 2—IR groups; 3—IR + Melatonin group; 4—IR + Sitagliptin group; 5—IR + Melatonin + Sitagliptin group; 6—IR + CC group; 7—IR + CC + Melatonin + Sitagliptin group	2 weeks	10 weeks	Improving antioxidative and antiapoptotic responses	Up-regulation of AMPK/SIRT1 activity via melatonin	[100]
Male C57BL/6 J mice	8 weeks	–	T1DM	T1DM 50 mg/kg (STZ) by intraperitoneal for 5 consecutive days + intraperitoneal injections of 10 mg/kg/d melatonin for 10 weeks	4 groups 1—normal glucose; 2—high glucose; 3—Mannitol; 4—melatonin	2 weeks	12 weeks after the first injection of STZ	amelioration of high glucose-induced CMECs injury by melatonin	Treatment of apoptosis and increased AMPK/SIRT1 signaling axis activity by melatonin in CMEC	[101]
Adult male Wister rats	8 weeks	170–200 (g)	T2DM	T2DM nicotinamide (100 mg/kg, ip) 20 min before STZ (55 mg/kg) + melatonin (10 mg/kg) by stomach tube daily for 15 days between 10:0 and 11:00 am	N = 20 4 groups (5 rats each) 1—control group received standard diet 2—melatonin treated group 3—diabetic group 4—group receiving melatonin for 15 days after the induction of diabetes	3 days	15 days post diabetic induction of STZ	Protective effects of melatonin against hyperglycemia, anti-lipid, antioxidant, anti-inflammatory and anti-apoptotic	MLT improves serum glucose levels, HbA1-c, lipid profile, insulin levels and insulin resistance, glutathione and IL-10 and Bcl-2 levels and prevents the increase of pro-inflammatory cytokines and the expression of Bax, caspase-3 and P53	[9]
Female Wistar strain rats	–	150–180 g	T1DM	T1DM 60 mg/kg (STZ) by intraperitoneal for 5 consecutive days + injected daily with melatonin i.p (10 mg/kg)	N = 30 3 groups (10 rats each) Group I; control non-diabetic rats; group II; STZ-induced, untreated diabetic rats; group III; STZ-induced, melatonin-treated diabetic rats	3 days	6 weeks post diabetic induction of STZ	The role of melatonin in controlling oxidative stress with its antioxidant properties	Bringing the levels of GSH, GSH-Px, and SOD closer to the control group in diabetic rats treated with melatonin	[23]
Male Wister rats	–	200–220 (g)	T1DM	T1DM 60 mg/kg (STZ) by intraperitoneal for 5 consecutive days + injected melatonin i.p (10 mg/kg) daily for 21 days at 11:00	N = 32 4 groups (8 rats each) I; control daily received intragastric administration of normal saline/ethanol; II; melatonin; III; diabetic; IV; diabetic + melatonin	2 days	21 days post diabetic induction of STZ	The positive effect of melatonin on diabetic myocardial damage and apoptosis	Increasing Bcl-2 expression and blocking activation of CD95 and caspases 9, 8, and 3 by oral melatonin treatment in diabetic	[16]
Male Sprague–Dawley rats	8–10-weeks	180–200 (g)	T1DM + T2DM	T1DM 60 mg/kg (STZ) by intraperitoneal + T2DM 0.125 mg/kg (dexamethasone solution) by subcutaneous administration during 13 days + Intraperitoneal injection (10 mg/kg) of melatonin from 14 to 23 days of the experiment	N = 42 5 groups I; control; II; T1DM; III; T2DM; IV; T1DM + melatonin; V; T2DM + melatonin	3 days	24 days	Melatonin as a positive regulator of the immune system	Reduction of TNF-α, IL-1β, and IL-6 mediated by melatonin	[1]
Mature male albino rats	–	0.18–0.20 kg	T1DM	Intraperitoneal injection of alloxan with 5% monohydrate solution at a dose of 170 mg/kg body weight + Intraperitoneal injection (10 mg/kg) of melatonin at 8 am for seven days after five days	N = 158 3 groups I; rats under artificial equinox; II; rats under constant dark; III; rats under constant light/ each group 5 subgroups: 1) control; 2) DM; 3) alloxan diabetic with melatonin; 4) alloxan diabetic with impaired glucose tolerance; 5) alloxan diabetic with IGT with melatonin	5 days	12 days	The positive effect of melatonin on impaired glucose tolerance under constant light conditions	Improvement of BG level and normalization of PK and LDH activities and increase of G6PD activity with melatonin administration	[10]
Male Sprague Dawley rats	8-weeks	–	T2DM	T2DM (a high-fat diet (40% fat, 41% carbohydrate, and 18% protein for 4 weeks) + induced with an intraperitoneal injection of STZ (60 mg/kg/day) + Oral induction of melatonin a dose of 20 mg/kg/day	N = 50 3 groups I; control (n = 15); II; DM (n = 20); III; DM + Mel (n = 20)	7 days	12 weeks	Amelioration of oxidative stress damage and apoptosis of diabetic aorta by melatonin	Activation of the Notch1/Hes1 signaling pathway by melatonin	[4]
Male Wistar rats	–	180–200 (g)	T1DM	DM (Intraperitoneal injection of 60 mg/kg of STZ) + receive daily 10 mg melatonin/kg/b.w. (i.p.)	N = 40 3 groups I; control; Injection of physiological solution containing 5% ethanol; II; DM; III; DM + Mel	3 days	18 days	Beneficial effects of melatonin in controlling vascular complications of diabetes	Prevention of increase in nitric oxide level in aortic tissue during diabetes with melatonin administration	[30]
Wild‐type mice	8‐weeks	20–25 (g)	T1DM	DM (intraperitoneal injection of STZ (50 mg/kg for 5 consecutive days)) + Oral administration of melatonin at a dose of 20 mg/kg per day for 4 weeks	N = 254 10 groups (a) wild type (n = 32); (b) melatonin (n = 32) (c) DM (n = 30); d) DM + melatonin (n = 30); (e) DM + Parkin (n = 24); (f) DM + Parkin + Mel (n = 25); (g) DM + Mst1 (n = 21); (h) DM + Mst1‐Tg (n = 20); (i) DM + Mst1 + Mel (n = 20); (j) DM + Mst1‐Tg + Mel (n = 20)	7 days	4 weeks	Melatonin rescues the impaired mitophagy activity of DCM	Melatonin enhances Mst1/Parkin-mediated mitophagy, thereby increasing clearance of dysfunctional mitochondria in mice with DCM	[102]
Sprague–Dawley rats	–	200–220 g	T2DM	HG treatment (500 g/L, 4 ml/kg/h, i.v.) + melatonin (10 mg/kg/d, i.p., 5 days before operation)	4 groups (1) Sham (2) MI/R + V (vehicle) (3) MI/R + HG (4) MI/R + HG + melatonin	–	–	protective effect of melatonin against myocardial ischemia–reperfusion (MI/R) injury in acute hyperglycemic state	Rescue of the thioredoxin system by melatonin through downregulation of Txnip expression by Notch1/Hes1/Akt signaling in a membrane receptor-dependent manner	[26]
Wild-type and SykCKO mice	8-weeks	-	T1DM	DM (intraperitoneally injected with STZ, 50 mg/kg for 5 consecutive days) + melatonin (20 mg/kg/d) for 12 weeks	8 groups 1; WT; 2; Syk; 3; Mel; 4; DM + WT; 5; DM + Syk; 6; DM + Mel; 7; DM + Ad; 8; DM + Mel + Ad	After the first 4 weeks	12 weeks	The role of melatonin in diabetic cardiomyopathy	Inactivation of Syk/COX-1/SERCA axis by melatonin treatment	[24]
Male Wistar rats	–	0.18–0.20 kg	T1DM	DM induced by injection of alloxan (170 mg/kg) by an i.p + Melatonin (10 mg/kg daily orally for 14 days from the fifth day)	2 groups Group I; DM; group II; DM + Mel	4 days	14 days post diabetic induction of STZ	Possible activation of glycolysis to restore events in the Cori cycle with melatonin	Restoration of pyruvate kinase activity and glycogen content to normal levels by melatonin in diabetic	[103]
Male Sprague–Dawley (SD) rats	–	250–280 (g)	T2DM	high-fat diet (containing 45% kcal as fat, 35% kcal as carbohydrate, and 20% kcal as protein) for 4 weeks and injection STZ (40 mg/kg, i.p) + receive melatonin at 10 mg/kg/d	(1) control; (2) diabetic; (3) diabetic with AAV9-NC and treated with or without melatonin; (4) diabetic with AAV9-SIRT6 shRNA and treated with melatonin; (5) diabetic subjected to sham surgery; (6) diabetic with negative control virus and treated with or without melatonin and then subjected to MI/R surgery; (7) diabetic with AAV9-SIRT6 shRNA and treated with melatonin and then subjected to MI/R surgery; (8) diabetic with luzindole and melatonin and then subjected to MI/R surgery	–	16 weeks post diabetic induction of STZ	A promising strategy to reduce DCM and reduce myocardial vulnerability to ischemia–reperfusion injury with melatonin	The pivotal role of melatonin in reducing myocardial vulnerability to MI/R injury with the focus of SIRT6-AMPK-PGC-1α-AKT	[104]
Male C57BL/6 J mice	–	–	T1DM	DM (intraperitoneally injected with STZ, 50 mg/kg for 5 consecutive days) + melatonin (10 mg/kg/d) for 4 weeks + H9c2 cells exposed to high glucose (33 mmol/L)	5 groups Group I; con; group II; DM; group III; DM + Mel; group IV; Sirt-1 + DM; group V; Sirt-1 + Mel + DM	2 weeks	12 weeks after the first injection of STZ	Prevention of mitochondrial fission to reduce diabetes-induced cardiac dysfunction with melatonin	Drp1-mediated attenuation of mitochondrial fission by melatonin in a SIRT1/PGC-1α-dependent manner	[28]
C57BL/6 wild-type mice	8–12 weeks	-	T1DM	T1DM (intraperitoneally injected with STZ, 50 mg/kg for 5 consecutive days) + melatonin (20 mg/kg/d) for 4 weeks	N = 80 4 groups Group I; Control; group II; Con + Mel; group III; DM; group IV; Mel + DM	5 days	3 months after the first injection of STZ	Regulating autophagy, limiting apoptosis, remodeling, and reducing cardiac dysfunction in DCM with melatonin	Mst1/Sirt3 signaling by melatonin	[77]
Male KM mice	–	20 ± 2 (g)	T1DM	T1DM (intraperitoneally injected with STZ, 60 mg/kg for 3 consecutive days) + melatonin (10 mg/kg/d)	N = 40 4 groups I; non-diabetes; II; DM; III; DM + Mel; IV; DM with 0.5% of ethanol solution treatment as a negative control	3 days	8 weeks after the first injection of STZ	Antifibrotic effect of melatonin for the treatment of DCM	Inhibition of lncRMALAT1/miR-141-mediated NLRP3 inflammasome activation and TGF-β1/Smads signaling by melatonin	[105]
Male Sprague–Dawley rats	–	180–200 (g)	T1DM	T1DM (intraperitoneally injected with STZ, 50 mg/kg for 3 consecutive days) + melatonin (10 mg/kg/d) for 5 days	N = 144; 4 groups (1) Con (n = 12); (2) DM (n = 12); (3) DM + Sham (n = 24); (4) DM + MI/R + vehicle treatment (n = 24); (5) DM + MI/R + MLT (n = 24); (6)DM + MI/R + MLT + KT5823 (n = 24); (7) DM + KT5823 (n = 12); (8) DM + MLT (n = 12)	7 days	5 days post diabetic induction of STZ	Amelioration of diabetic MI/R damage and reduction of myocardial apoptosis and oxidative stress to maintain cardiac function with melatonin	Modulation of Nrf-2-HO-1 and MAPK signaling by melatonin in diabetic MI/R injury	[106]
Male Wistar albino rats	–	250–300 (g)	T1DM	DM (intraperitoneally injected with STZ, 60 mg/kg) + melatonin (10 mg/kg/d) for 8 weeks + control received 0.1 M citrate buffer + 6 U/kg/day NPH insulin	N = 48 6 groups (1) Con (n = 8); (2) Mel (n = 8); (3) DM (n = 8); (4) DM + Mel (n = 8); (5) DM + insulin (n = 8); (6) DM + insulin + MLT (n = 8)	48 h	8 weeks after the first injection of STZ	The therapeutic role of melatonin and insulin in preventing the damage caused by diabetes	Improving contractile responses and restoring responses to acetylcholine and reducing oxidative stress with melatonin and insulin treatment	[25]
Male mice	–	25–30 (g)	T1DM	DM (intraperitoneally injected with STZ, 50 mg/kgW) + melatonin (3 mg/kg/d) twice a week for consequent four weeks	N = 40 4 groups (n = 10) Control group (C), Control group + melatonin (CM), Diabetic group (D), Diabetic + melatonin (DM) group	3 days	4 weeks post diabetic induction of STZ	Effects of melatonin on aging factors with age to reduce cardiac damage in hyperglycemic conditions	Reversal of increased β-galactosidase and suppression of SOX2, Klotho, and Telomerase genes in T1D mice by melatonin administration	[107]
Male mice	–	25–30 (g)	T1DM	DM (intraperitoneally injected with STZ, 50 mg/kgW) + melatonin (3 mg/kg/d) twice a week for consequent four weeks + Swimming exercises for four weeks	N = 50 5 groups (n = 10) Control; Diabetic group; Diabetic + Melatonin group; Diabetic + Exercise group; and Diabetic + Exercise + Melatonin group	3 days	4 weeks post diabetic induction of STZ	Reducing the harmful effects of diabetes on heart tissue with exercise and melatonin	Increase of cardiac SOD, GPx with the decrease of MDA and increase of TAC and decrease of TNF-α, caspase-3, and suppression of expression of Connexin-43 and Sirtuin1 in the combination of exercise and melatonin	[17]
male Wistar rats	12 weeks	270–340 g	T1DM	T1DM 45 mg/kg of STZ by intraperitoneal + Exercise protocol (Motorized rodent treadmill with electric shock plate motivation for 8 weeks, 5 days per week/ In the first 4 weeks, increasing the duration and speed of training gradually from 30 to 60 min per day and from 18 m per minute to 24 m per minute with a constant slope of 10 degrees during the study and a 2-min rest at the end of the training and no change in the parameters Exercise until the end of the study/ Fixed placement of sedentary mice without exercise on the treadmill)	N = 48 4 groups (12 rats each) i) Sedentary control, ii) sedentary diabetic, iii) exercise control, iv) exercise diabetic	3 days	8 weeks	Prevention of cardiac autonomic neuropathy by early initiation of systemic exercise training	favorable change in the balance between parasympathetic and sympathetic activity	[54]
Male Wistar rats	–	350–500 g	T1DM	T1DM 50 mg/kg of STZ by intraperitoneal + Treadmill exercise protocol once a day, five days a week, for nine weeks/ The first week of animal adaptation (8 min, 8 m per minute)/ In the second week, increasing the duration and speed of training gradually up to 18 min a day at a speed of 11 m per minute/ Start training in the first two weeks with low voltage electrical stimulation	N = 79 sedentary control (C-Sed, *n* = 14); exercised control (C-Ex, *n* = 15); sedentary diabetes (DM-Sed, *n* = 25); and exercised diabetes (DM-Ex, *n* = 25)	7 days	8 weeks	Reduction of left atrial dilatation and myocardial oxidative stress and dysfunction with low-intensity exercise	Decreasing the diameter of the left atrium and improving the function of the papillary muscles and increasing the activity of Antioxidant enzymes	[55]
Wistar male rats	–	250–270 g	T1DM	T1DM 50 mg/kg of STZ by intraperitoneal + Voluntary exercise of mild/moderate intensity in cages equipped with vertical treadmills for 24 h a day	nine groups (n = 10): 1- Diabetic sham castration + placebo group, 2-Diabetic + placebo group, 3-Diabetic + Testosterone group, 4-Diabetic + Exercise + placebo group, 5-Diabetic + Exercise + Testosterone group, 6-Diabetic + castrated + placebo group, 7-Diabetic + castrated + Testosterone group, 8-Diabetic + castrated + Exercise + placebo group,9-Diabetic + castrated + Testosterone + Exercise group	72 h	6 weeks	Preventing the progression of diabetic cardiomyopathy due to angiogenesis in the heart by exercise	Increased expression of miRNA-126 in heart tissue	[56]
Wistar male rats	Four months old	230—250 g	T1DM	T1DM 50 mg/kg of STZ by intraperitoneal + Voluntary exercise of mild/moderate intensity in cages equipped with vertical treadmills for 24 h a day	N = 63 1—Diabetes: 2—Diabetes—Testosterone 3—Diabetes– Exercise 4—Diabetes -Exercise—Testosterone 5—Diabetes—castrated 6—Diabetes—castrated—Testosterone 7—Diabetes—castrated -Exercise 8—Diabetes—castrated – Testosterone-Exercise	72 h	6 weeks	Heighten the body's antioxidant system with exercise	Increasing the activities of SOD, GPX, and CAT and decreasing the level of MDA	[36]
C57BL/6J mice	10-week	20–25 g	T2DM	The normal diet contained 17% kcal from fat and 3.1 kcal/g + Training with moderate intensity on the treadmill and gradually increasing the speed and duration of running for five days a week	four groups: (1) normal diet, (2) ND mice exercise, (3) HFD, (4) HFD-exercise	2 weeks	20 weeks	Exercise modulating hydrogen sulfide and pyroptotic signaling in the heart	Increasing cardiac H2S concentration and expression of H2S biosynthesis enzymes and protecting the diabetic heart by reducing pyroptosis with exercise	[43]
Male Wistar rats	–	200–250 g	T1DM	T1DM 50 mg/kg of STZ by intraperitoneal + Volunteer training for 24 h a day for 6 weeks	N = 28 four groups (n = 7): control, exercise, diabetes, and exercise + Diabetes	–	6 weeks	Voluntary exercise is a useful tool to reduce oxidative stress in diabetes	Decreased MDA levels and increased SOD, GPX, and CAT levels	[33]
Sprague–Dawley rats	16–8 weeks	–	T1DM	T1DM 120 mg/kg of Alloxan by intraperitoneal + Treadmill exercise in the control and diabetic groups at a speed of 18 m per minute, 40 min per day for 5 days per week	N = 40 four groups (n = 10): sedentary control, exercised control, sedentary diabetic rats, and exercised diabetic rats	3 days	8 weeks	Improvement of cardiac VEGF expression due to diabetes with treadmill exercise training	Increased expression of VEGF	[64]
Male diabetic db/db mice	4-week	–	T2DM	Moderate intensity treadmill training 5 days a week for 8 weeks (Week 1 running for 10 min at 10 m/min, 20 min at 10 m/min for week 2, 30 min at 12 m/min for week 3 weeks 4 to 8, 30 min at 15 m/min)	1-sedentary (db/db-sedentary) 2-exercise-trained (db/db-exercise) group	2-week	8 weeks	Improvement of cardiac markers of angiogenesis and endothelial dysfunction	A higher percentage of total HB and HB1AC and a decrease in TNF-α protein expression of TNF-α and mRNA expression of IL-6 and IL-1β	[41]
Male Wistar rats	–	210–230 g	T1DM	T1DM 60 mg/kg of STZ by intraperitoneal + running wheels equipped with digital wheel distance counters for 60 days	N = 32 4 groups: Control Sedentary(n = 6), Diabetic Sedentary(n = 10), Control Running(n = 6) and Diabetic Running(n = 10)	10 days	60 days	Reluctance to participate in voluntary exercises and no significant effect of exercise on diabetic heart function	Decreased glucose levels with exercise and less mileage in diabetic rats	[108]
Male Sprague Dawley rats	4–6 months	180–200 g	T2DM	T2DM (a high-fat diet for 28 days + injected intraperitoneally 35 mg /kg) of STZ) + Swimming exercises for 5 min in the first week and a gradual increase for 5 days a week for 4 weeks	N = 32 4 equal groups; a) normal control, b) DM, c) DM + Exercise, d) DM + stevia R extracts	48 h	4 weeks	Cardioprotective effects of exercise against DCM	The effect of exercise on the concentration of MDA and catalase enzyme and the concentration of glutathione	[38]
Male Sprague Dawley rats	6-week	400–600 g	T2DM	T2DM (a high-fat high-sugar diet for 7 weeks + injected STZ (30 mg/kg, i.p) + control group inject citrate buffer (0.25 ml/kg)) + Aerobic exercise protocol: A motor-driven treadmill (In the first three days, the speed of the treadmill is 5 min at a speed of 8 m per minute and then change to 10 min at a speed of 10 m per minute) + MOTS-c treatment protocol: injected (0.5 mg/kg/day, i.p.), for 7 days/week	N = 55 1-control (C, n = 10) 2-high-fat high-sugar diet plus STZ (n = 45), 2–1- diabetes, 2–2-diabetes exercise, 2–3-diabetes plus MOTS-c treatment	3 days	8 weeks	Exercise-induced cardio-protection in diabetes	Activation of NRG1-ErbB4 signaling	[109]
Male Wistar rats	–	250–300 g	T1DM	T1DM 60 mg/kg of STZ by intraperitoneal + running exercise on a treadmill (5 days/week, 60 min/day at 22 m/min, 0-degree slope), at 10:00 AM, for 8 weeks + IMODTM (20 mg/kg) injected intraperitoneally, once a day at 8:00 AM for 8 weeks	8 groups (n = 8): control, exercise, IMODTM, exercise + IMODTM, diabetes, diabetic + exercise, diabetic + IMODTM, diabetic + exercise + IMODTM	72 h	8 weeks	Positive effects of exercise on oxidative stress and markers of heart damage and increasing the activity of antioxidant enzymes	The positive effects of exercise on the reduction of MDA and LDH along with the increase of TAC, SOD, and glutathione peroxidase	[37]
Male Wistar rats	8 Weeks	200–250 g	T1DM	Intraperitoneal injection of nicotinamide solution with a dose of 120 mg/kg and after 15 min STZ with a dose of 65 mg/kg + Endurance training, 5 sessions per week for 20–30 min with a speed of 27 m/min and an intensity of 75% of VO2max in the first week and a gradual increase to 60 min with a speed of 27 m/min and an intensity of 75% of VO2max	N = 36 3 groups 1—Endurance training, 2-—Diabetic control group and 3—Healthy control group	1 week	10 weeks	The positive effect of endurance training on angiogenesis and improvement of diabetic heart	Increased VEGF and VEGFR2 gene expression	[62]
Female Wistar rats	–	249–253 g	T1DM	T1DM 50 mg/kg of STZ by intraperitoneal + Treadmill acclimatization (10 min/day; 0.3 km/h) for 1 week/motorized treadmill training at low intensity (50% to 70% of maximum running speed) for 1 h/day, 5 days/week for 8 weeks, with a gradual increase in speed from 0.3 to 1.2 km/h	N = 52 Sedentary control (n = 8), trained control (n = 8), sedentary diabetic (n = 20), and trained diabetic (n = 16)	72 h	11 weeks after STZ injection	Improved autoregulation induced by exercise training	Exercise improves baroreflex sensitivity and heart rate and increases vagal tone	[110]
Male Wistar rats	–	200 ± 217 g	T1DM	T1DM 55 mg/kg of STZ by intraperitoneal + exercise program for 4 weeks (5 sessions per week) at a speed of 15 to 18 m/min for 25 to 44 min	N = 40 control, diabetes, control + exercise and exercise + Diabetes	2 weeks	4 weeks	Reduction of apoptotic complications in diabetic cardiomyocytes with exercise	Decreased NT-proBNP	[86]
Male Wistar rats	10–12 Weeks	200–250 g	T1DM	T1DM 30 mg/kg of STZ by intraperitoneal + Aerobic exercise program with the intensity of 50–60% VO2max, 5 days a week for 6 weeks	N = 19; 4 groups: training(n = 6), sham(n = 6), control(n = 4) and healthy(n = 3)	4 days	6 weeks	Improving inflammatory indices and diabetic heart damage with exercise	Significant decrease in TNF-α and CK and a significant increase in PGC-1α	[40]
Male Wistar rats	8–10 weeks	253–265 g	T1DM	T1DM 50 mg/kg of STZ by intraperitoneal + Empagliflozin 10 mg/kg daily by oral gavage for six weeks + Endurance training program on the treadmill with a zero-degree slope for 5 sessions per week and 10 min at a speed 10 m per minute in the first week and the sixth week for 30 min at a speed of 18 m per minute in each training session	N = 40 five groups: control, diabetic, diabetic + empagliflozin, diabetic + training and diabetic + training + empagliflozin	2 weeks	6 weeks	Aerobic exercise improves the inflammatory status, structure, and function of diabetic heart tissue	Decreased TNF-α and TGF-β	[58]
Male Wistar rats	–	200–232 g	T1DM	T1DM 60 mg/kg of STZ by intraperitoneal + ET on a treadmill daily for 8 weeks + GSE (200 mg/kg) orally via gavage once a day	N = 45 five groups: sedentary control, sedentary diabetic, trained diabetic, GSE-treated secondary diabetic, and GSE-treated trained diabetic	1 day	8 weeks	The effect of exercise on improving left ventricular dysfunction	Improvement of systolic pressure gradient related to diastolic pressure	[111]
Male Wistar rats	6–8 weeks	140 ± 10 g	T1DM	T1DM 50 mg/kg of STZ by intraperitoneal + Acute resistance training includes four training sessions (climbing a 1-m ladder with a 2-cm net ladder and weights attached to the rats' tails + first day, 10 climbs without weight bearing + second day, light weights of 0.2 to 0.5 weights body + third day, 4–6 repetitions with weights of 0.2 to 0.5 of own body weight + increasing the weights gradually (30 g) + fourth day doing 10 climbs with 70 to 75% of your maximum carrying capacity with a 1.5-min rest in between repetitions	N = 20 two groups: (1) acute resistance exercise (2) sedentary control	4 days	4 days	The effect of resistance exercise on oxidative stress	Decreases MDA	[112]
Male outbred Wistar rats	12-week	280–320 g	T1DM	T1DM 60 mg/kg of STZ by intraperitoneal + Treadmill exercise training with gradual increases in speed and time running up to 1.8 km/h, 1.5 h/d, 5 days a week for 8 weeks	N = 34 untrained (n = 15) and trained (n = 19) groups	2 weeks	8 weeks	Prevention of adverse effects of diabetes on antioxidant defense with aerobic exercise	Decreased GPX activity	[113]
male Wistar rats	–	300 ± 350 g	T1DM	T1DM 60 mg/kg of STZ by intraperitoneal + control groups intraperitoneal injection of an equal volume of citrate buffer + treadmill exercise 5 days a week for an hour with 22 (m/min) speeds	6 groups (n = 10): sedentary control, control with 15-day exercise, control with 60-day exercise, sedentary diabetic, diabetic with 15-day exercise, and diabetic with 60-day exercise	48 h	5 days	Prevention of diabetic heart hypertrophy with exercise	A decrease in the average ratio of heart weight to body weight	[59]
male Wistar rats	10 Weeks	220 ± 20 g	T1DM	T1DM 60 mg/kg of STZ by intraperitoneal + control group same volume of citrate buffer + aerobic training for 12 weeks in 5 sessions per week with a gradual increase in speed (18–26 m/min) and 10 to 55 min in the form of running on the treadmill	N = 21 Three groups: diabetic aerobic training, diabetic and healthy control groups	72 h	12 weeks	Improving heart function and preventing diabetic heart diseases with exercise	Increase expression Akt1 and mTORc1 genes	[114]
Old male wild-type mice + homozygous (db/db,C57BLKS/J)	5 weeks	–	T2DM	treadmill running 5 days/week, 60 min/day at a final intensity equivalent to approximately 50% of VO2Max	Two groups Diabetic + exercise or Diabetic + sedentary treatments	–	10 weeks	Improving endothelial function and reducing chronic inflammation with exercise training	Reduction of IL-6, TNF-α protein level and improvement of insulin sensitivity and up-regulate SOD and phosphorylated- eNOS protein expression	[78]
Male Sprague–Dawley rats	4 weeks	125–150 g	T2DM	a high-fat diet (40% fat, w/w) and a low-dose of streptozotocin (35 mg/kg/ body mass^)–1^) by intravenous injection + voluntary wheel running	a sedentary group or an exercise-trained group	24 h	12-week	Prevention of diabetic cardiomyopathy and disruption of SR protein content regulation by exercise	Improvement of SERCA2a protein content and maximum SERCA2a activity (Vmax)	[115]
Male Wistar rats	–	200–220 g	T1DM	T1DM 55 mg/kg of STZ by intraperitoneal + diabetes groups receive 0.9 IU × 100 g − 1 insulin once a day for 6 weeks + Running at speed of 18 m/min with a slope of 5%, for 30 min, once a day on a treadmill	N = 24 (n = 6): 1. diabetic 2. Insulin diabetic 3. Exercise + diabetic 4.Insulin + exercise + diabetic	48 h	6 weeks	Protection of heart diseases due to diabetes by exercise	Better effect on NDNF and VEGF	[116]
Male C57BL/6 mice	8 week	–	T2DM	Intraperitoneal injection of glucose solution (1.5 g/Kg) + treadmill running once per day, five times a week for 4 weeks at 60% of their maximal aerobic velocity	1-control (n = 35) fed standard diet 2- group received a high fat/high sucrose diet, 2–1: sedentary (n = 45), 2–2: exercise (n = 35)	12 weeks	4 weeks	Cardioprotective effect of regular exercise on diabetic heart vulnerability	Decreased iNOS expression and nitro-oxidative stress	[117]
CD1 male mice	10–12 weeks	25–35 g	T2DM	STZ injections over a 5-days (Low Dose STZ protocol) + Swimming training consists of 2 sessions a day with a 4-h rest period starting with 10 min and gradually increasing by 10 min daily for 5 days a week for 6 weeks	3 groups, a sedentary non-diabetic group, a sedentary diabetic group, and a swim-trained diabetic group	1 week	6 weeks	Beneficial effects of exercise on improving diabetic heart function	Decrease intracellular protein *O*-GlcNAcylation	[118]
db/db mice	–	–	T2DM	The db/ + control and db/db group of mice were exercised on a treadmill with a controlled speed (7 m/min. for db/db mice and 10 m/min. for db/ + controls) for 300 m/day, 5 days/week	4 groups: (i) db/ + control, (ii) db/ + control + exercise, (iii) db/db (iv) db/ db + exercise	–	8 weeks	Reduction of fibrosis and myocyte detachment in diabetic heart	Decreased MMP9 activity in the diabetic exercise group	[119]
Male Sprague–Dawley rats	12 weeks	250–300 g	T1DM	Intraperitoneal injection of STZ (40 mg/kg) + treadmill exercise protocol (30 min daily for 4 weeks at a speed of 10 m/min)	N = 24 (each group = 8) 3 groups: A (control), B (diabetic untreated), and C (diabetic treated with low-intensity exercise)	3 days before to STZ	4 weeks	Reducing oxidative stress and apoptosis and maintaining myocardial integrity with low-intensity exercise	Decrease in myofibril loss, vacuolation of cytoplasm, and irregularity of fibrils and decrease in MDA and increase in SOD, GSH-Px, and CAT	[35]
C57BL/6 male mice	6–8 Weeks	180–200 g	T1DM	Injected intraperitoneally STZ (50 mg/kg body weight per day for 5 consecutive days) + treadmill exercise regularly at a speed of 22 cm/sec for 60 min per day, 5 days a week	N = 20 (a) sedentary, (b) exercised, (c) diabetes, (d) diabetic + exercise	1 week	12 weeks	Exercise inhibits cardiac remodeling in DCM	Inhibited Mst1 and miR-486a5p release	[60]
Male Sprague–Dawley rats	–	8-week	T2DM	A high-fat diet of total energy 20 kJ/g + intraperitoneally injected STZ at 30 mg/kg BW + NC group (intraperitoneal injection of 0.01 mM citric acid buffer of equal volume) + 8 weeks of exercise at moderate intensity by the speed of 15.2 m/min, the slope of 3°, 60 min per day, and 5 days per week	1-control (n = 8) 2-T2DM (n = 16) 3- T2DM + aerobic treadmill exercise (n = 16)	72 h	8 weeks	Exercise as an alternative therapy for diabetic cardiomyopathy	Suppressing expression of MMP-2, CTGF, TGF-β1, p-Smad2 and p-Smad3, and increased expression of TIMP–1, Smad7	[57]
Male Wistar rats		200–250 g	T1DM	intraperitoneal injection of STZ 50 mg/kg + voluntary exercise + testosterone 2 mg/kg/day	9 groups (n = 7): 1, sham operation; 2, diabetic; 3, testosterone; 4, exercise; 5, testosterone + exercise; 6, castrated; 7, testosterone–castrated; 8, exercise–castrated; 9, testosterone and exercise–castrated	2 days	6 weeks	Improving angiogenesis by exercise in diabetic rats	Enhancement miR-132 levels	[120]
Male C57BL/6 mice	6 weeks	–	T2DM	Injected intraperitoneally low-dose STZ (120 mg/kg body weight) + a high-fat diet (45% of energy as fat) + exercise by training at 0.5/0.6/0.7/0.8/1.0 km/h for 1 h	1—non-diabetes Mellites sedentary control, 2—STZ/HF sedentary control, 3—STZ/HF treadmill running,	1 week	16 weeks	The effect of exercise in improving diabetic cardiomyopathy	Improving blood pressure and systolic dysfunction and increasing the level of oxidative phosphorylation, increasing the membrane potential and reducing the level of ROS and oxygen consumption	[57]
Male Sprague Dawley	6-week	–	T2DM	T2DM = 7 weeks by a high-fat diet combined with a low-dose injection of STZ (30 mg/kg) + Control rats with vehicle citrate buffer (0.25 ml/kg) + Aerobic Exercise Protocol- A motor-driven treadmill for aerobic exercise training (a speed of 21 m/min for 1 h, 50–60% of VO2 max (1 h per days for 5 days of 8 weeks)) + Resistance Exercise Protocol (A special animal ladder 1 m long, with 2 cm grid steps and an 85° gradient)	Six groups (1) non-diabetic sedentary control, (2) non-diabetic aerobic exercise control, (3) non-diabetic resistance exercise control, (4) diabetic sedentary control, (5) diabetic aerobic exercise, (6) diabetic resistance exercise	3 days	8 weeks	Improving diabetic heart function with aerobic exercise	Increase the expression levels of titin and decrease collagen I, TGFβ1 expression level	[121]
Male Wistar rats	12–14 weeks	140–180 g	T1DM	Intraperitoneal injection of nicotinamide (110 mg/kg body weight) and STZ (50 mg/kg body weight) + training group in an 8-week exercise protocol on a treadmill with an intensity of 25 m per minute, a slope of 5% and 30 min per session	Two groups: diabetic control and diabetic training	48 h	8 weeks	Aerobic exercise as an activator of the angiogenic pathway of diabetic heart tissue	Increase expression mir-126, raf1, PI3K, VEGF and decrease blood glucose levels and insulin resistance	[122]
Male Sprague‐Dawley	–	200 ± 20 g	T1DM	T1DM = a high‐fat and high‐ Sugar diet for 4 weeks + intraperitoneal injections STZ twice (40 mg/kg) + Control (a regular chow and injection with the same citrate buffer) + Running for 60 min on 5 days a week on a treadmill with an incline of 10 degrees/treadmill speed in the LIT group (20 m/min) and the HIT group (34 m/min)	n = 40 1- diabetic cardiomyopathy, 2-DCM + low‐intensity training, 3-DCM + high‐intensity training, 4- control	5 weeks	12 weeks	Improvement of diabetic cardiomyopathy with exercise	Enhances cardiac IGFI-R/PI3K/Akt and Bcl-2 family-associated pro-survival pathways	[46]
Male Wistar rats	8-weeks	–		+ moderate aerobic exercise training on a treadmill 60 min/day, 5 days/week, for 10 weeks	N = 48 control, diabetes, DM + exercise	48 h	10 weeks	The effect of exercise training on cardiac survival pathways in diabetic rats	Enhances cardiac IGFI-R/PI3K/Akt and Bcl-2 family associated pro-survival pathways	[45]

BG: blood glucose; CAT: Catalase; Con: Control; DM: Diabetes mellitus; GPx: Glutathione peroxidase; HFD: High Food Diet; HG: high glucose; IL-1β: Interleukin 1 beta; IL-6: Interleukin 6; LDH: Lactate dehydrogenase; MDA: Malondialdehyde; Mela: melatonin; PK: Pyruvate kinase; SOD: Superoxide dismutase; STZ: Streptozocin; T1DM: Type 1 Diabetes mellitus; T2DM: Type 2 Diabetes mellitus; TAC: total antioxidant capacity; TNF-α: Tumor necrosis factor α; VEGF: Vascular endothelial growth factors

### Risk of bias in the included studies

In the current study, a modified CAMARADES quality checklist was used to assess the internal and external validity of the selected studies. The checklist contains details notably randomized allocation (model/sham groups), blinded induction of the model and assessment of outcomes, calculation of the sample size, compliance with the existing animal welfare act, the disclosure of all relevant conflicts of interest, reporting of animal exclusions, and publication in peer-reviewed journals. All articles had been issued in peer-reviewed journals.

## Results

### Mel-treated diabetic rodents

#### Oxidative status

**Fig. 2:**
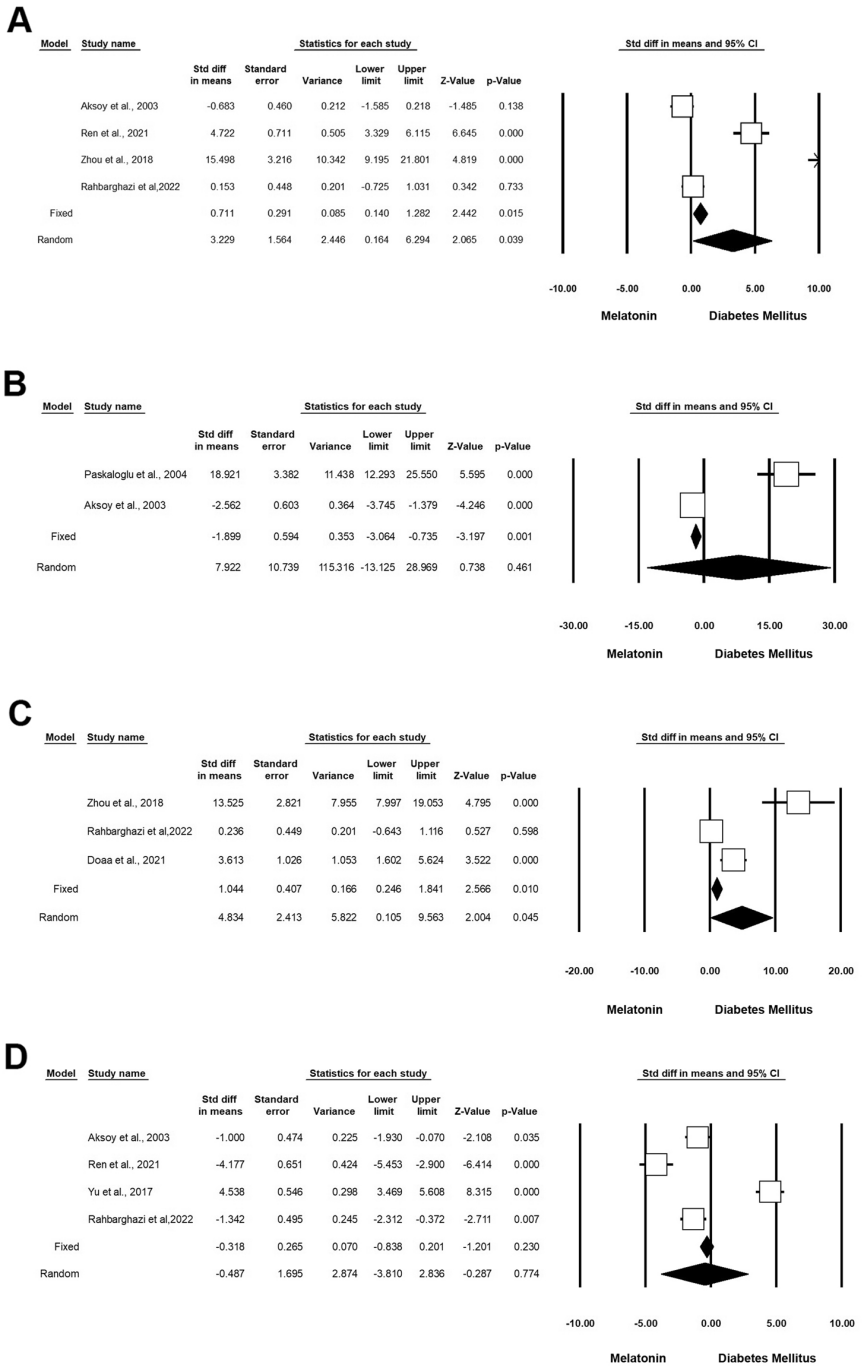
Improvement of oxidative status in diabetic rodents with Mel administration. CI: confidence interval. **A** Superoxide dismutase, **B** glutathione, **C** glutathione peroxidase, **D** malondialdehyde

According to our analysis, administration of Mel on diabetic rodents can significantly improve antioxidant capacity [superoxide dismutase (SOD), glutathione, and glutathione peroxidase (GPx)] compared to the matched control groups. Based on the data, 4 experiments (n = 198; 41 Mel + DM and 41 DM) were associated with the impact of Mel on SOD under diabetic conditions. These studies indicated that Mel can significantly induce the activity of SOD in diabetic rats (Standardized mean difference (SMD): 3.229 CI 95%, 0.164 to 6.294; p = 0.039; I^2^ = 95.29%) (Fig. 2A) [4, 17, 23, 24]. Two experiments (n = 78; 18 Mel-treated DM and 18 DM) studied the effect of Mel on glutathione under diabetic conditions. Heterogeneity analysis indicated p < 0.001 and Higgins’ I^2^ reached 97.44%. Results showed that in the random model, SMD for GSH was 7.922 (CI 95%, − 13.125 to 28.969; p = 0.461) (Fig. 2B) [23, 25]. Along with these studies, 3 experiments (n = 106; 21 Mel + DM and 21 DM) investigated the therapeutic effects of Mel on GPx activity under diabetic conditions. Heterogeneity analysis and Higgins’ I^2^ were p < 0.001 and 93.12%, respectively. The analysis results showed SMD of GPx was 4.834 (CI 95%, mean difference: 0.105 to 9.563; p = 0.045; Fig. 2C) [9, 17, 24]. Other 4 experiments (n = 226; 59 Mel + DM and 59 DM) released data associated with the impact of Mel on malondialdehyde (MDA) under diabetic conditions. Heterogeneity analysis for these experiments yielded a p-value of < 0.001 and Higgins’ I^2^ reached 97.51%. Therefore, the random model was applied and the results showed that SMD of MDA reached − 0.487 (CI 95%, mean difference: − 3.810 to 2.836; p < 0.774) (Fig. 2D and Table 3) [4, 17, 23, 26].

**Table 3:** Forest plot results of the effect of Mel on diabetic heart tissue

Outcome	Number of studies	Effect size and 95% interval			Test of null (2-Tail)				Heterogeneity	
		Point estimate	Lower limit	Upper limit	Z-value	P-value	Q-value	df (Q)	P-value	I-squared
SOD	4	3.22	0.164	6.29	2.06	0.039	63.74	3	< 0.001	95.29
GSH	2	7.92	− 13.12	28.96	0.73	0.461	39.10	1	< 0.001	97.44
GPX	3	4.83	0.10	9.56	2.004	0.045	29.09	2	< 0.001	93.125
MDA	4	− 0.48	− 3.81	2.83	− 0.28	0.774	120.62	3	< 0.001	97.51
Caspase3	6	− 3.730	− 7.84	0.38	− 1.77	0.076	189.45	5	< 0.001	97.36
Plasma glucose	6	− 4.54	− 7.05	− 1.85	− 3.36	0.001	88.98	5	< 0.001	94.38
Bcl-2	4	3.64	− 0.70	8.00	1.64	0.101	108.68	3	< 0.001	97.24
Bax	3	− 2.20	− 9.19	4.78	− 0.61	0.536	124.53	2	< 0.001	98.39
Apoptosis index	4	− 8.61	− 11.94	− 5.28	− 5.06	< 0.001	21.58	3	< 0.001	86.10
Total cholesterol	4	− 5.44	− 8.19	− 2.70	− 3.88	< 0.001	22.27	3	< 0.001	86.53
Triglyceride (mg/dL)	4	− 6.60	− 11.05	− 2.15	− 2.90	0.004	57.03	3	< 0.001	94.74
HDL	4	5.97	0.70	11.24	2.22	0.026	77.28	3	< 0.001	96.11
VLDL	4	− 7.95	− 13.62	− 2.29	− .275	0.006	57.36	3	< 0.001	94.77
IL-6	3	− 5.46	− 14.02	3.09	− 1.25	0.211	61.33	2	< 0.001	96.73
TNF-a	3	− 11.57	− 21.92	− 1.22	− 2.19	0.028	31.51	2	< 0.001	93.65
IL-1β (ng/ml)	2	− 55.60	− 156.32	45.12	− 1.08	0.279	17.42	1	< 0.001	94.26
p53	2	− 10.32	− 13.28	− 7.37	− 6.84	< 0.001	1.57	1	0.210	36.36

#### Inflammatory status

**Fig. 3:**
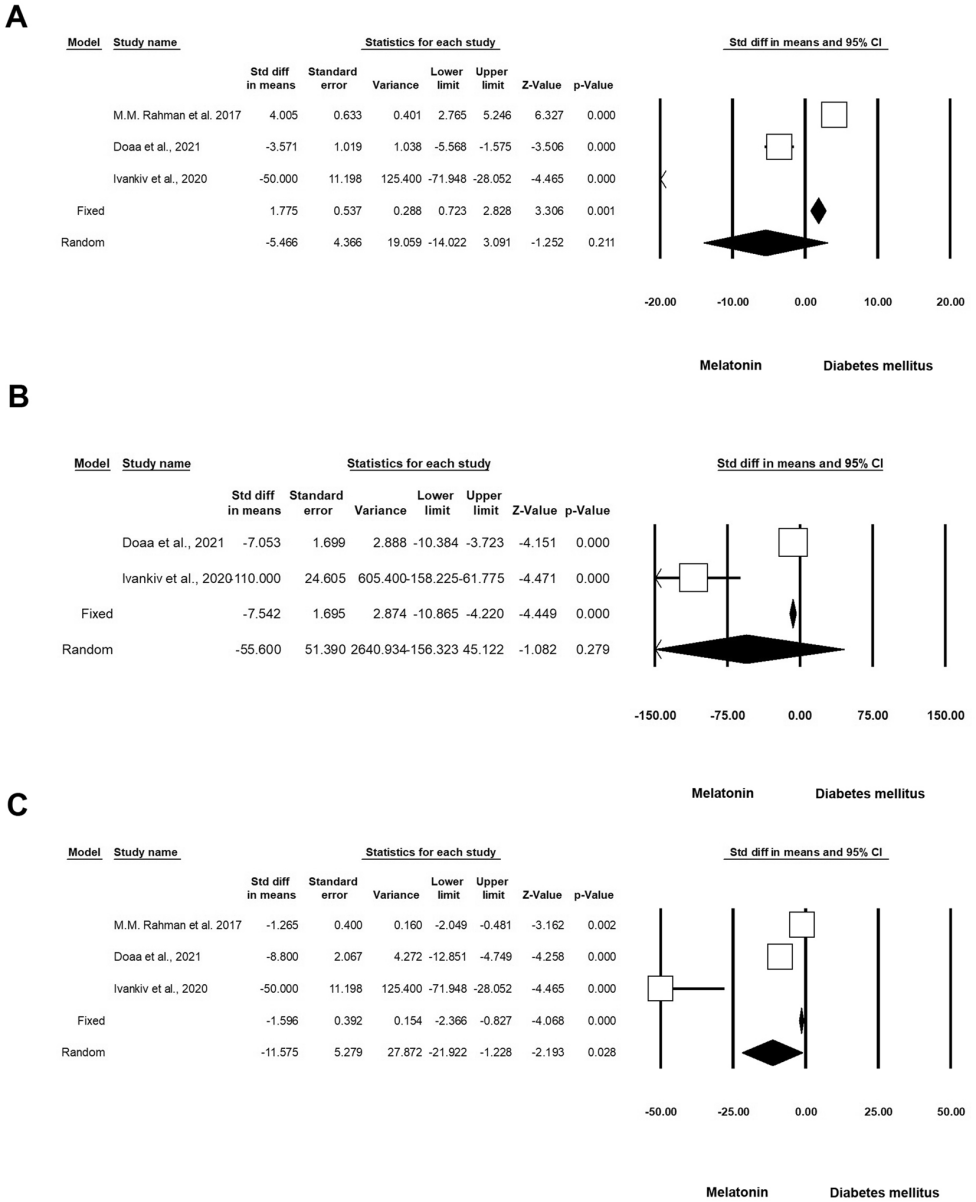
Modulation of inflammatory status in diabetic rodents received Mel. CI: confidence interval. **A** IL-6, **B** IL-1β, **C** TNF-α

According to our data, 3 experiments (n = 110; 25 Mel + DM and 25 DM) investigated the effect of Mel on interleukin-6 (IL-6) levels in diabetic rats. Heterogeneity analysis revealed a significant difference (p = 0.001) between control and diabetic rats with Higgins’ I^2^ values of 96.73%. The results showed that in the random-effect model, the SMD of IL-6 was -5.466 (CI 95%, mean difference: − 14.022 to 3.091; p = 0.211) (Fig. 3A) [1, 9, 27]. Besides, 2 experiments (n = 35; 10 Mel + DM diabetic and 10 DM) monitored the changes in the levels of IL-1β in diabetic rats after administration of Mel. Based on data, heterogeneity, and Higgins’ I^2^ were p < 0.001 and 94.26%, respectively. The SMD of IL-1β was − 55.600 (CI 95%, mean difference: -156.323 to 45.122; p = 0.279) (Fig. 3B) [1, 9]. There are 3 experiments (n = 110; 25 Mel + DM and 25 DM) related to the effect of Mel on TNF-α in diabetic rats. Heterogeneity analysis revealed a p-value of < 0.001 and Higgins’ I^2^ score was 93.65%. Using the random-effect model, the SMD of TNF-α was − 11.575 (CI 95%, mean difference: − 21.922 to − 1.228; p = 0.028; Fig. 3C and Table 3) [1, 9, 27].

#### Apoptotic indices

**Fig. 4:**
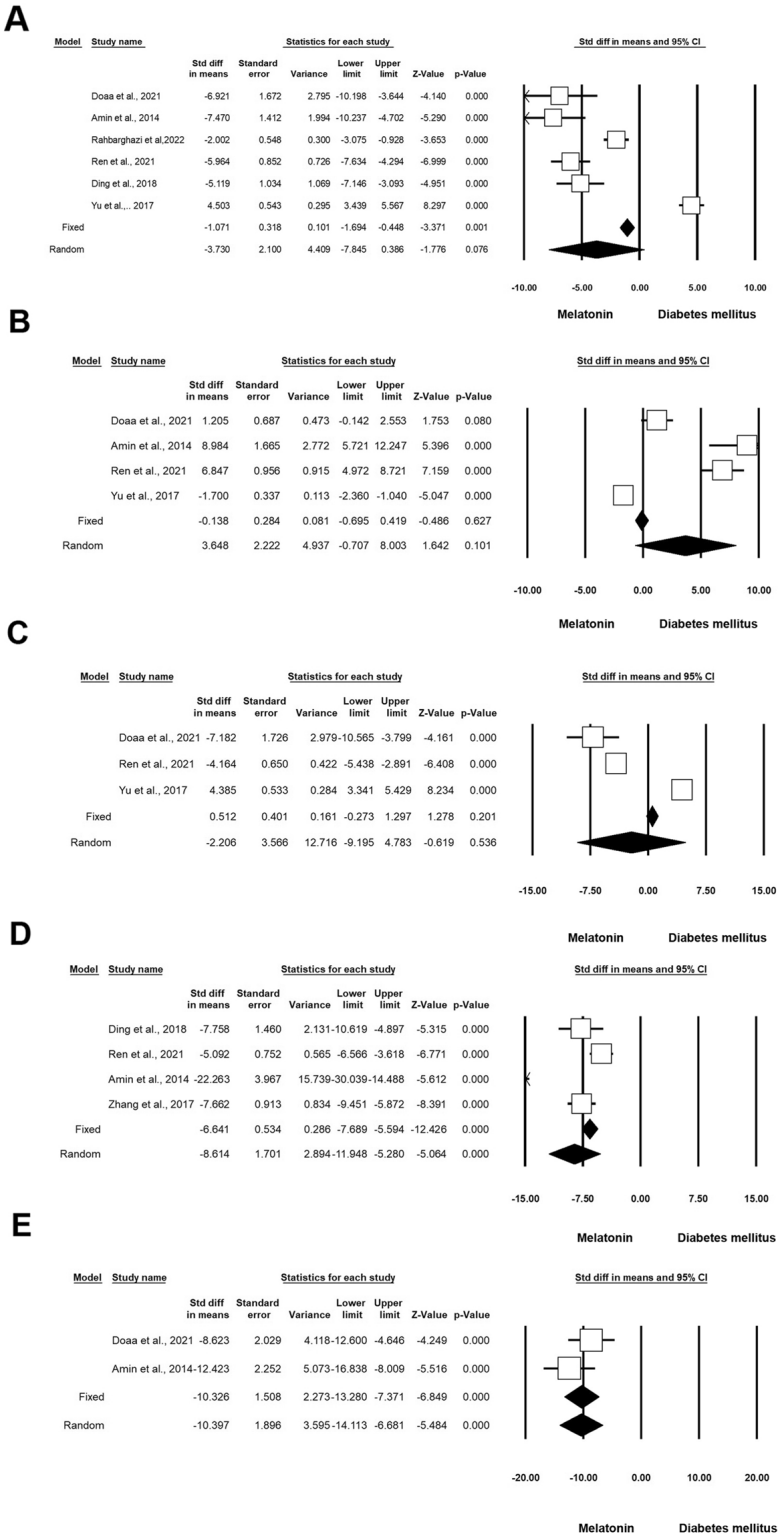
Status of Caspase-3 in diabetic rodents received Mel. CI: confidence interval. **A** Caspase-3, **B** Bcl-2. **C** Bax, **D** Apoptosis Index, **E** P53

Six experiments (n = 288; 70 Mel + DM and 70 DM) were associated with the effect of Mel on Caspase-3 activity under diabetic conditions. Heterogeneity analysis indicated p < 0.001 and Higgins’ I^2^ was 97.36%. When using the random-effect model, the SMD of Caspase-3 was − 3.730 (CI 95%, − 7.845 to 0.386; p = 0.076; Fig. 4A) [4, 9, 16, 17, 26, 28]. We also found that four experiments (n = 198; 52 Mel-treated DM and 52 DM) released data on the status of Bcl-2 in diabetic conditions after administration of Mel. Heterogeneity analysis showed a p-value of < 0.001 and Higgins’ I^2^ was 97.24%. SMD of Bcl-2 was 3.648 (CI 95%, − 0.707 to 8.003; p = 0.101) in the random model (Fig. 4B) [4, 9, 16, 26]. Three experiments (n = 166; 44 Mel-treated diabetics and 44 diabetics) were found related to the analysis of Bax levels under diabetic conditions and Mel administration. Heterogeneity analysis indicated that the p-value and Higgins’ I^2^ were < 0.001 and 98.39%, respectively. The SMD of Bax was -2.206 (CI 95%, − 9.195 to 4.783; p = 0.536; Fig. 4C) [4, 9, 26] in the analysis according to the random-effect model. Four experiments (n = 162; 51 Mel-treated diabetics and 51 diabetics) were conducted to evaluate the apoptosis index under diabetic conditions with Mel administration. According to heterogeneity analysis, the p-value and Higgins’ I^2^ were < 0.001 and 86.10%, respectively. The apoptosis index was reduced after intervention according to the random model analysis (SMD: − 8.614 (CI 95%, − 11.948 to − 5.280; p < 0.001; Fig. 4D) [4, 16, 28, 29]. Two experiments (n = 52; 13 Mel-treated diabetics and 13 diabetics) investigated the levels of p53 in diabetics subjected to Mel administration. Heterogeneity analysis indicated p = 0.210 and Higgins’ I^2^ value of 36.36%. Based on the low heterogeneity analysis, the SMD of p53 was − 10.326 (CI 95%, − 13.280 to − 7.371; p < 0.001) in the fixed-effect model, and this value reached -10.397 (CI 95%, − 14.113 to − 6.681; p < 0.001) in the random model (Fig. 4E and Table 3) [9, 16].

#### Lipid and Glucose profiles

**Fig. 5:**
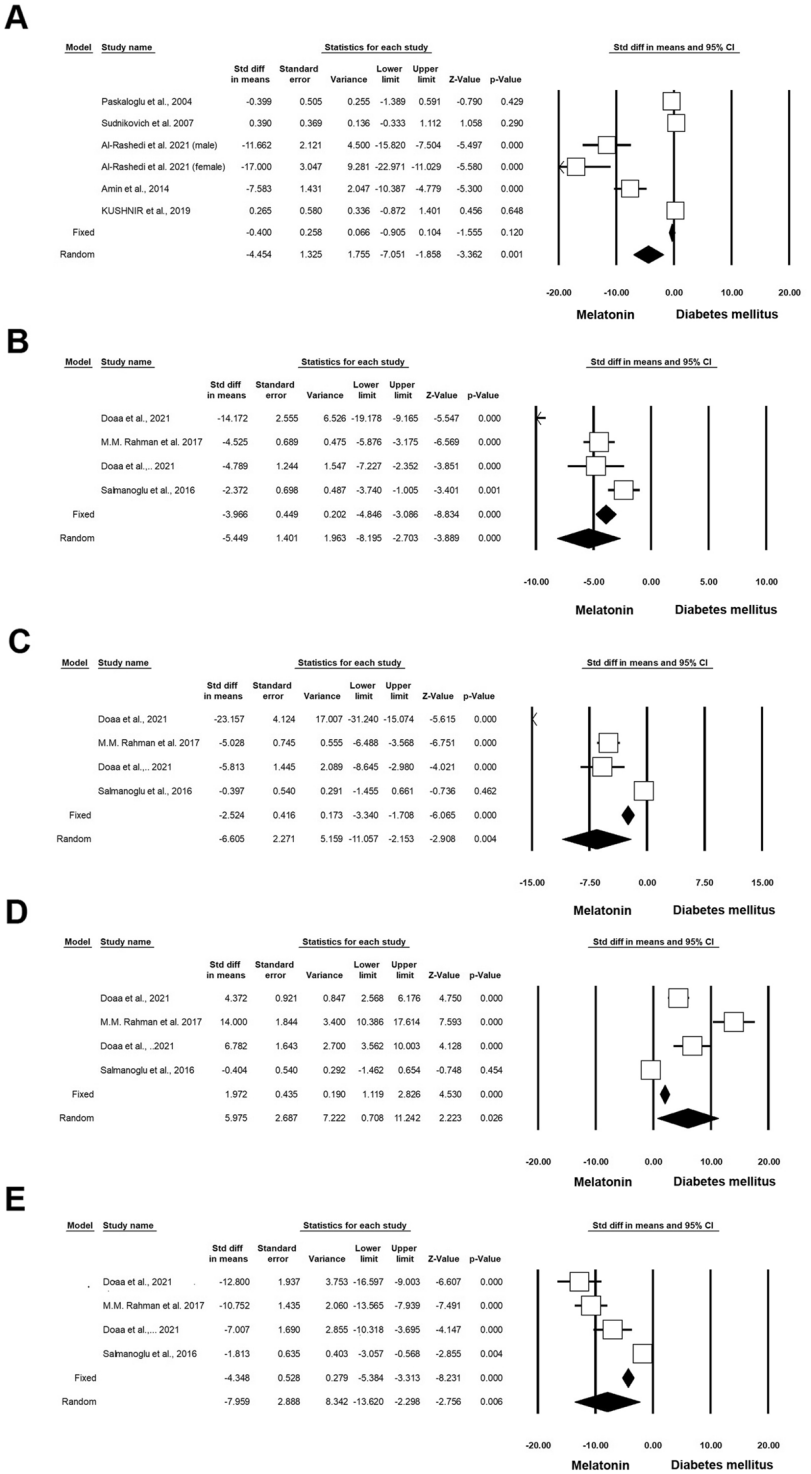
Glucose levels in in diabetic rodents received Mel. CI: confidence interval. **A** Level Glucose, **B** total cholesterol, **C** triglyceride, **D** HDL, **E** vLDL

Six experiments (n = 218; 53 Mel + DM and 53 DM) were associated with the effect of Mel on lipid and glucose profiles under diabetic conditions. Heterogeneity analysis revealed that the p-value and Higgins’ I^2^ were 0.001 and 94.38%, respectively. The results showed that the SMD of glucose level was − 4.454 (CI 95%, − 7.051 to − 1.858; p < 0.001; Fig. 5A) [10, 16, 25, 30, 31]. Four experiments (n = 156; 35 Mel + DM and 35 DM) were done concerning total cholesterol (TC) analysis in DM with the administration of Mel. Heterogeneity analysis indicated P = 0.001 and Higgins’ I^2^ was 86.53%. TC was reduced after treatment (SMD: − 5.449; CI 95%, − 8.195 to − 2.703; p < 0.001; Fig. 5B) [9, 27, 32]. Four experiments (n = 156; 35 Mel + DM and 35 DM) released data on triglyceride in DM and Mel. Heterogeneity analysis indicated p < 0.001 and Higgins’ I^2^ was 94.74%. Similar to TC, the amount of TG was decreased (SMD:-6.605; CI 95%, − 11.057 to − 2.153; p = 0.004; Fig. 5C) [9, 27, 32]. Four experiments (n = 156; 35 Mel + DM and 35 DM) released data on HDL in diabetic rodents that received Mel. Heterogeneity analysis indicated p = 0.001 and Higgins’ I^2^ was 96.11%. The HDL value was increased in treated rats with Mel using the random model analysis (SMD: 5.975; CI 95%, 0.708 to 11.242; p = 0.026; Fig. 5D) [9, 27, 32]. Four experiments (n = 156; 35 Mel + DM and 35 DM) released data on VLDL in DM and Mel. Heterogeneity analysis indicated p < 0.001 and Higgins’ I^2^ was 94.77%. Data showed that the amount of VLDL was decreased in the random model (SMD: 5.975; CI 95%, − 13.620 to − 2.298; p = 0.006; Fig. 5E and Table 3) [9, 27, 32].

### Exercise-treated diabetic

#### Oxidative status

**Fig. 6:**
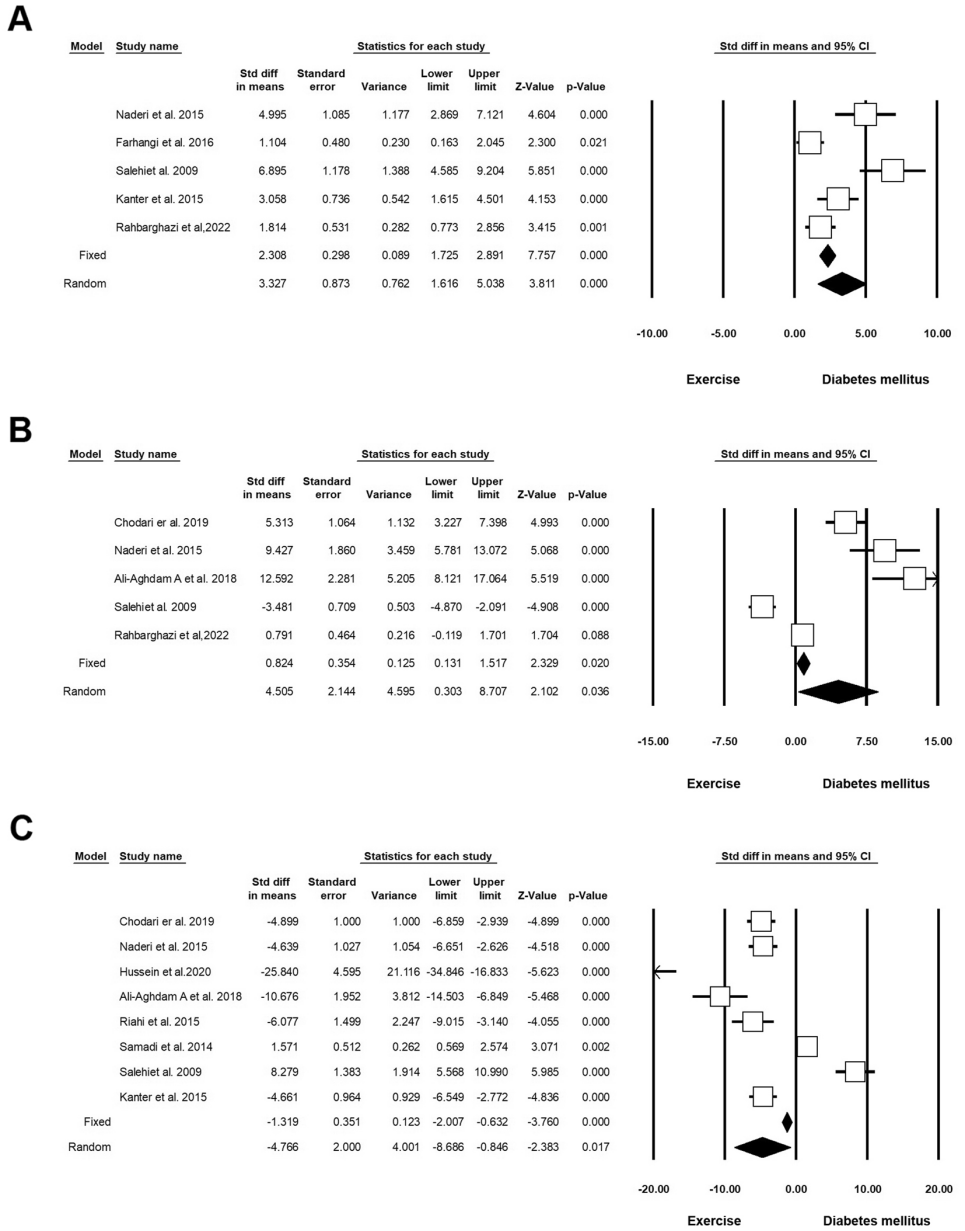
Oxidative stress status in diabetic rodents with regular exercise. CI: confidence interval. **A** Superoxide dismutase (SOD), **B** glutathione peroxidase (GPx), **C** malondialdehyde (MDA)

Five experiments (n = 162; 45 Exc + DM and 45 DM) released data on SOD under diabetic conditions exposed to Exc. Heterogeneity analysis indicated p = 0.001 with Higgins’ I^2^ value of 86.42%. SMD of SOD was 3.327 (CI 95%, 1.616 to 5.038; p < 0.001; Fig. 6A) [17, 20, 33–35] using a random effect model. Five experiments (n = 156; 43 Exc + DM and 43 DM) released data on GPx in DM and Exc. Heterogeneity analysis indicated p < 0.001 and Higgins’ I^2^ was 96.10%. SMD of GPx in the random model was 4.505 (CI 95%, 0.303 to 8.707; p = 0.036; Fig. 6B) [17, 33, 34, 36, 37]. Eight experiments (n = 204; 64 Exc + DM and 64 DM) released data on MDA in DM and Exc. Heterogeneity analysis indicated p = 0.001 and Higgins’ I^2^ was 96.04%. The SMD of MDA was − 4.766 (CI 95%, − 8.686 to − 0.846; p = 0.017; Fig. 6C and Table 4) [17, 33, 34, 36–40] in the random effect model.

**Table 4:** Forest plot results of the effect of exercise on diabetic heart tissue

Outcome	Number of studies	Effect size and 95% interval			Test of null (2-Tail)		Heterogeneity			
		Point estimate	Lower limit	Upper limit	Z-value	P-value	Q-value	df (Q)	P-value	I-squared
SOD	5	3.32	1.61	5.03	3.81	< 0.001	29.47	4	< 0.001	86.42
GPX	5	4.50	0.30	8.70	2.10	0.036	102.65	4	< 0.001	96.10
MDA	8	− 4.76	− 8.68	− 0.84	− 2.38	0.017	176.87	7	< 0.001	96.04
Caspase3	5	− 11.45	− 16.58	− 6.33	− 4.38	< 0.001	100.85	4	< 0.001	96.03
Plasma glucose	12	− 2.77	− 3.77	− 1.78	− 5.50	< 0.001	79.59	11	< 0.001	86.18
Bcl-2	5	2.92	− 0.93	6.77	1.48	0.138	77.49	4	< 0.001	94.83
Bax	5	− 1.62	− 6.78	3.53	− 0.61	0.536	117.49	4	< 0.001	96.59
Apoptosis index	3	− 6.53	− 11.64	− 1.41	− 2.50	0.012	21.64	2	< 0.001	90.76
Total cholesterol	2	− 7.56	− 9.21	− 5.91	− 8.98	< 0.001	0.037	1	0.848	0
Triglyceride (mg/dL)	2	− 3.40	− 5.19	− 1.62	− 3.75	< 0.001	3.71	1	0.054	73.06
HDL	2	9.28	− 1.35	19.92	1.71	0.087	25.84	1	< 0.001	96.13
VLDL	2	− 6.92	− 10.03	− 3.82	− 4.37	< 0.001	4.00	1	0.045	75.05
VEGF	5	3.69	1.83	5.55	3.89	< 0.001	33.46	4	< 0.001	88.04
IL-6	2	− 1.26	− 8.63	6.11	− 0.33	0.738	36.98	1	< 0.001	97.29
TNF-a	2	− 11.86	− 15.05	− 8.66	− 7.27	< 0.001	0.005	1	0.994	0
IL-1β (ng/ml)	2	− 5.20	− 13.12	2.72	− 1.28	0.198	19.69	1	< 0.001	94.92
p53	4	− 6.34	− 13.74	1.04	− 1.68	0.093	94.09	3	< 0.001	96.81

#### Inflammatory status

**Fig. 7:**
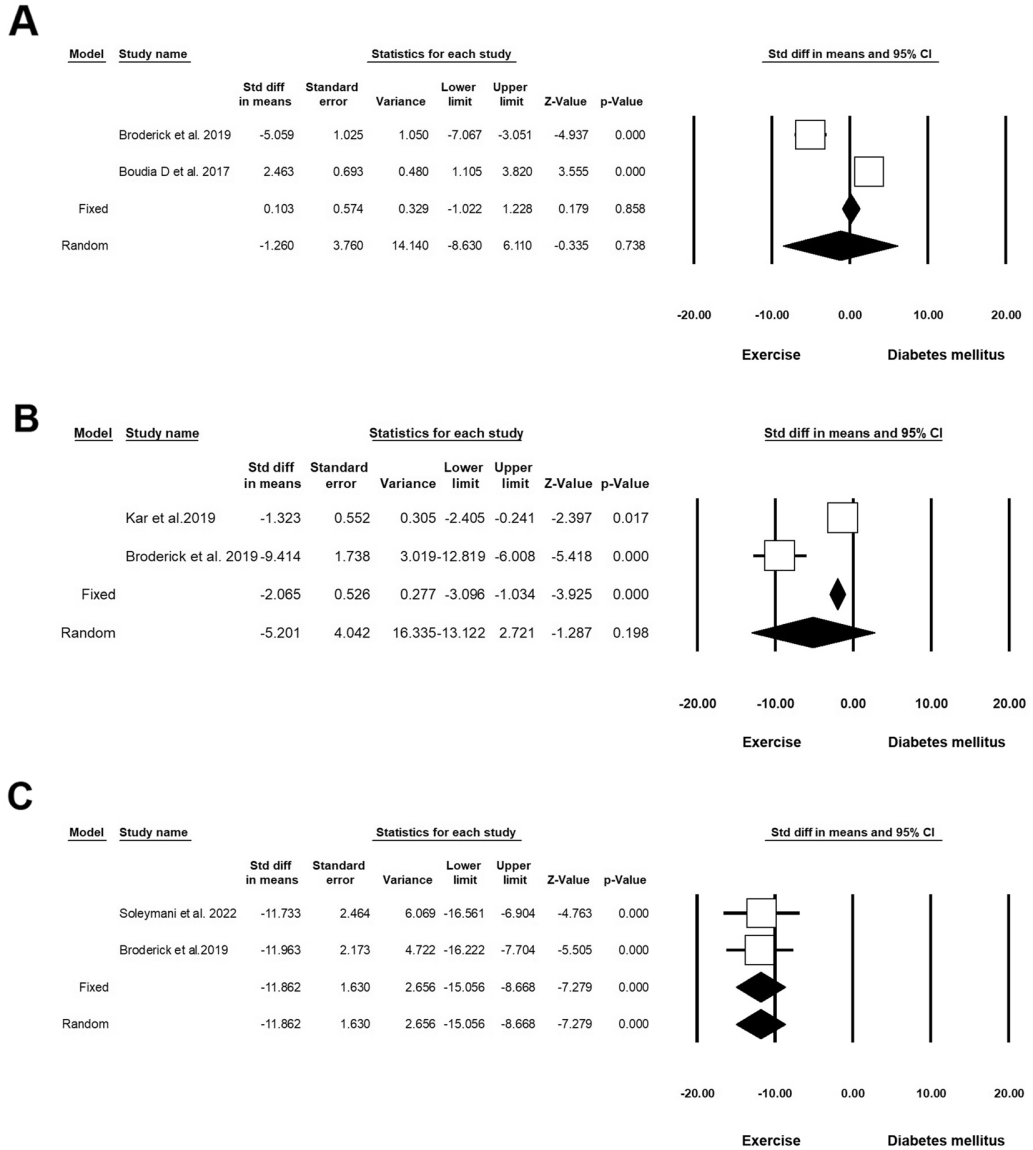
Inflammatory response status in diabetic rodents with regular exercise. CI: confidence interval. **A** IL-6, **B** IL-1β, **C** TNF-α

Two experiments (n = 72; 17 Exc + DM and 14 DM) released data on IL-6 in DM and Exc. Heterogeneity analysis indicated p < 0.001 and Higgins’ I^2^ was 97.29%. Analysis results showed that in the random model, the SMD was − 1.260 (CI 95%, − 8.630 to 6.110; p = 0.738; Fig. 7A) [41, 42]. Two experiments (n = 56; 16 Exc + DM and 16 DM) released data on IL-1β in DM and Exc. Heterogeneity analysis indicated p < 0.001 and Higgins’ I^2^ was 94.92%. The analysis results showed a decrease in IL-1β in the random model (SMD: − 5.201 (CI 95%, − 13.122 to 2.721; p = 0.198; Fig. 7B) [41, 43]. Two experiments (n = 39; 14 Exc + DM and 14 DM) released data on TNF-α in DM and Exc. Heterogeneity analysis indicated p = 0.994 and Higgins’ I^2^ was 00.00%. Analysis results showed that in the fixed-effect model, SMD of TNF-α was − 11.862 (CI 95%, − 15.056 to − 8.668; p < 0.001), and in the random model this value reached − 11.862 (CI 95%, − 15.056 to − 8.668; p < 0.001; Fig. 7C and Table 4) [41, 44].

#### Apoptotic indices

**Fig. 8:**
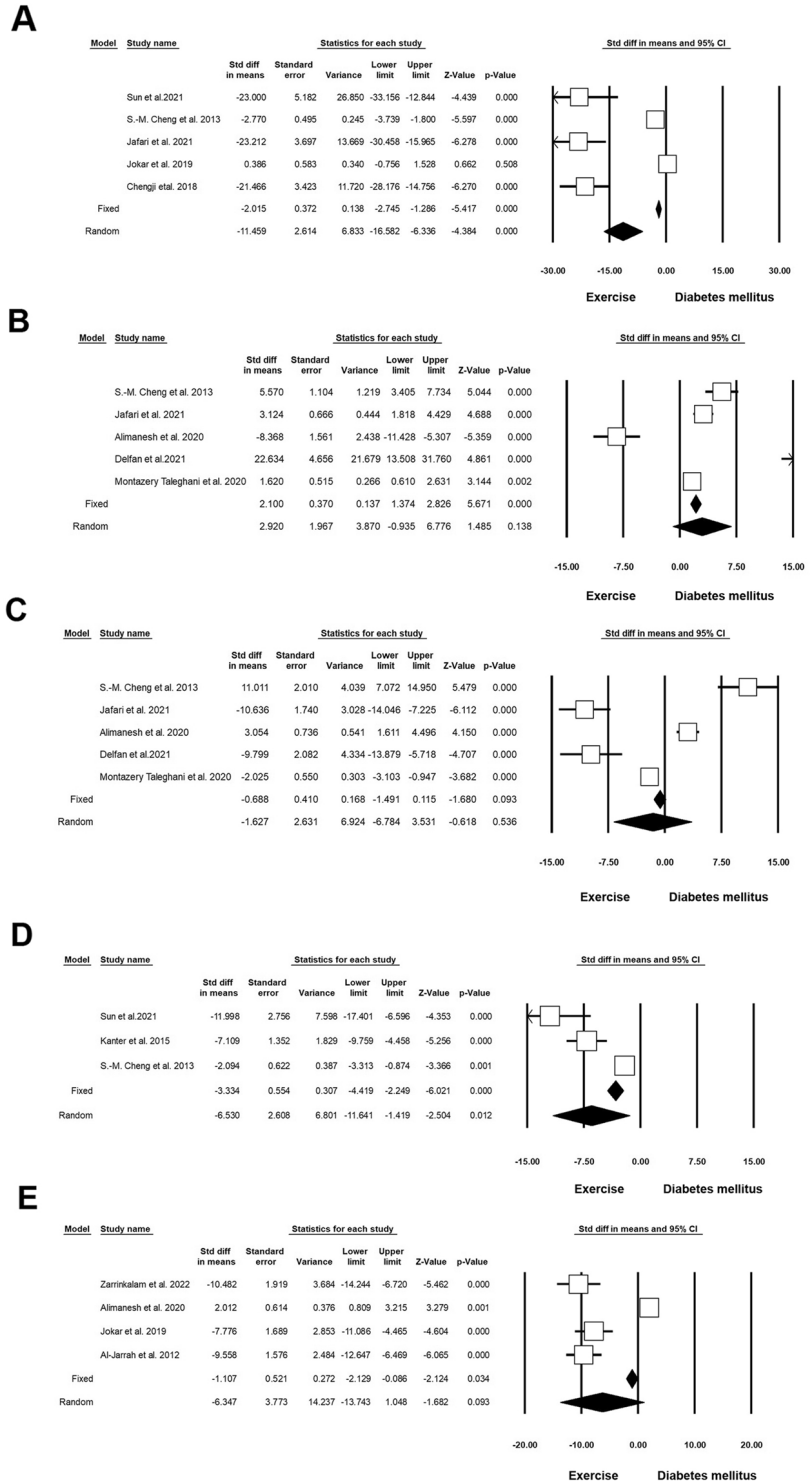
Apoptotic indices in diabetic rodents with regular exercise. CI: confidence interval. **A** Caspase-3, **B** Bcl-2, **C** Bax, **D** Apoptosis Index, **E** P53

Five experiments (n = 140; 47 Exc + DM and 47 DM) released data on Caspase-3 in DM and Exc. Heterogeneity analysis indicated p < 0.001 and Higgins’ I^2^ was 96.03%. In the random model, Caspase-3 was decreased (SMD: − 11.459; CI 95%, − 16.582 to − 6.336; p < 0.001; Fig. 8A) [45–49]. Five experiments (n = 110; 42 Exc + DM and 42 DM) released data on Bcl-2 in DM and Exc. Heterogeneity analysis indicated p < 0.001 and Higgins’ I^2^ was 94.83%. The SMD of Bcl-2 was 2.920 (CI 95%, − 0.935 to 6.776; p = 0.138; Fig. 8B) [45, 48, 50–52] in random effect analysis. Five experiments (n = 110; 42 Exc + DM and 42 DM) released data on Bax in DM and Exc. Heterogeneity analysis indicated p < 0.001 and Higgins’ I^2^ was 96.59%. The value of Bax was decreased in the intervention group compared to the non-treated control (SMD: -1.627; CI 95%, 6.924 to 3.531; p = 0.536; Fig. 8C) [45, 48, 50–52]. Three experiments (n = 68; 21 Exc + DM and 21 DM) released data on the apoptosis index in DM and Exc. Heterogeneity analysis indicated p < 0.001 and Higgins’ I^2^ was 90.76%. SMD of apoptosis index was reduced in the experiment group in the random model (SMD: − 6.530; CI 95%, − 11.641 to − 1.419; p = 0.012; Fig. 8D) [35, 45, 49]. Four experiments (n = 100; 32 Exc + DM and 32 DM) released data on p53 in DM and Exc. Heterogeneity analysis indicated p < 0.001 and Higgins’ I^2^ was 96.81%. Analysis results showed that the SMD of p53 in the random model was -6.347 (CI 95%, -13.743 to -1.048; p = 0.093; Fig. 8E and Table 4) [47, 48, 50, 53].

#### Lipid and Glucose profiles

**Fig. 9:**
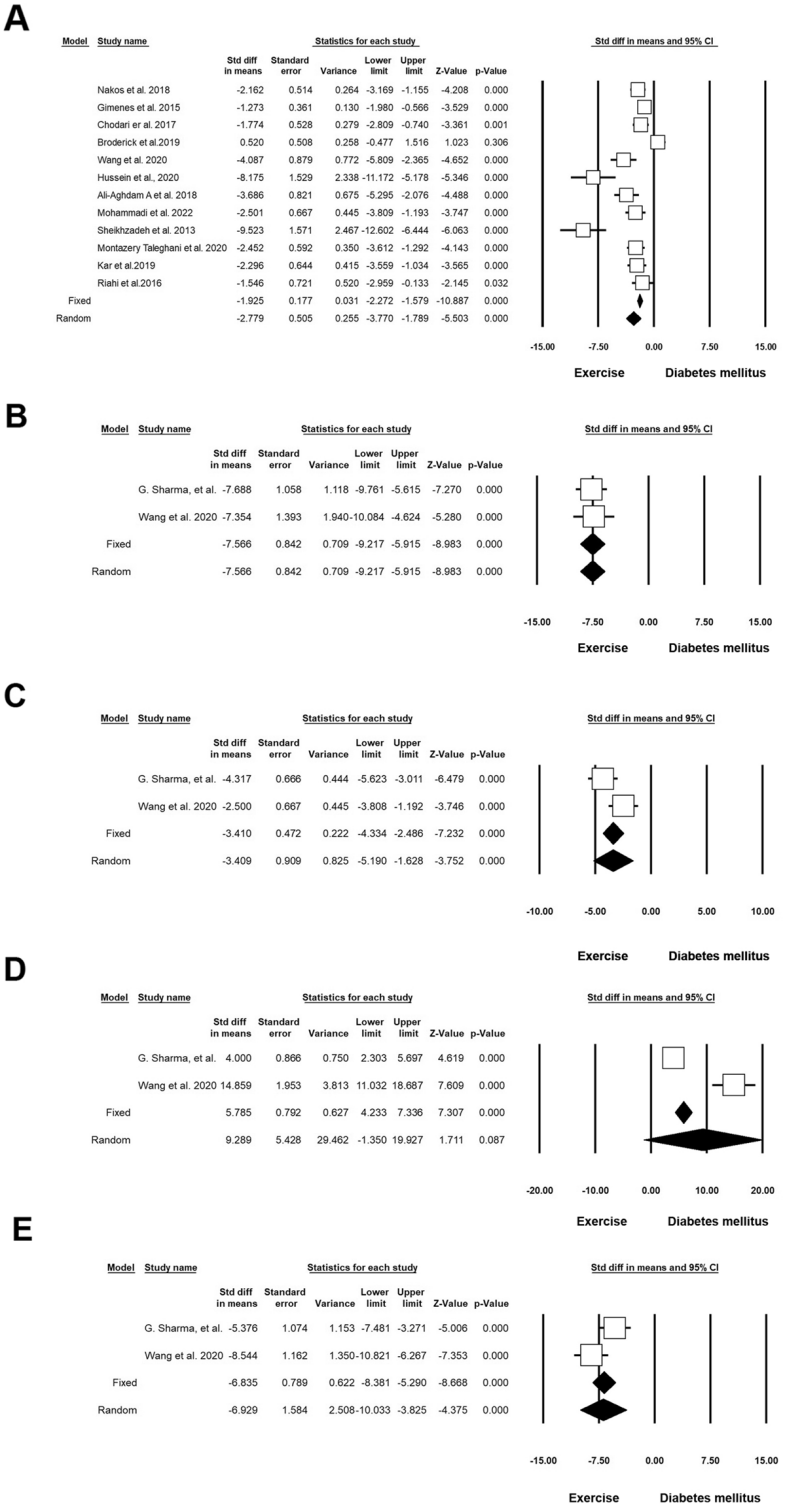
Improvement of lipid and glucose profiles in diabetic rodents with regular exercise. CI: confidence interval. **A** Level glucose, **B** total cholesterol, **C** triglyceride, **D** HDL, **E** vLDL

Twelve experiments (n = 362; 113 Exc + DM and 114 DM) released data on the level of glucose in DM and Exc. Heterogeneity analysis indicated p < 0.001 and Higgins’ I^2^ was 86.18%. Analysis results showed that SMD of glucose levels in the random model was -2.779 (CI 95%, -3.770 to -1.789; p < 0.001; Fig. 9A) [37–39, 41, 43, 52, 54–59]. Two experiments (n = 84; 23 Exc + DM and 23 DM) released data on TC in DM and Exc. Heterogeneity analysis indicated p = 0.848 and Higgins’ I^2^ was 00.00%. Results showed that in the fixed-effect model, the SMD of TC was − 7.566 (CI 95%, − 9.217 to − 5.915; p < 0.001), and in the random model this value reached − 7.566 (CI 95%, − 9.217 to − 5.915; p < 0.001; Fig. 9B) [57, 60]. Two experiments (n = 84; 23 Exc + DM and 23 DM) released data on triglyceride levels in DM and Exc. Heterogeneity analysis indicated p = 0.054 and Higgins’ I^2^ was 73.06%. Analysis results showed that in the fixed-effect model, the SMD of triglycerides was − 3.410 (CI 95%, − 4.334 to − 4.466; p < 0.001) and in the random model this value reached − 3.409 (CI 95%, − 5.190 to − 1.628; p < 0.001; Fig. 9C) [57, 60]. Two experiments (n = 84; 23 Exc + DM and 23 DM) released data on HDL in DM and Exc. Heterogeneity analysis indicated p < 0.001 and Higgins’ I^2^ was 96.13%. Results showed that HDL was increased in the random model (SMD: 9.299; CI 95%, − 1.350 to 19.927; p < 0.087; Fig. 9D) [57, 60]. Two experiments (n = 84; 23 Exc + DM and 23 DM) released data on VLDL in DM and Exc. Heterogeneity analysis indicated p = 0.045 and Higgins’ I^2^ was 75.05%. Analysis results showed that in the fixed-effect model, SMD of VLDL was − 6.835 (CI 95%, − 8.381 to − 5.290; p < 0.001), and in the random model, this value reached − 6.929 (CI 95%, − 10.033 to − 3.825; p < 0.001; Fig. 9E and Table 4) [57, 60].

#### VEGF

**Fig. 10:**
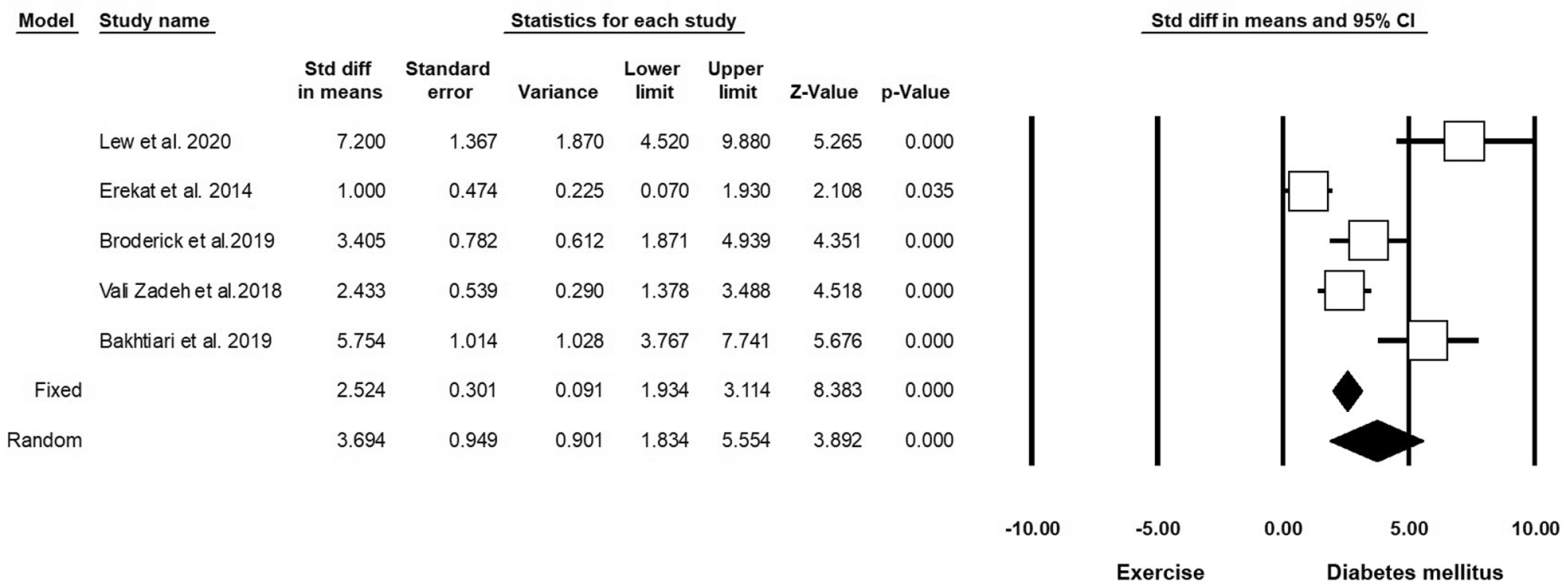
Changes in VEGF levels in diabetic rodents with regular exercise. CI: confidence interval

Five experiments (n = 162; 48 Exc + DM and 48 DM) released data on VEGF in DM and Exc. Heterogeneity analysis indicated p = 0.210 and Higgins’ I^2^ was 36.36%. Subgroup analysis results showed that in the fixed-effect model, SMD of VEGF was 2.524 (CI 95%, 1.934 to 3.114; p < 0.001), and in the random model this value was 3.694 (CI 95%, 1.834 to 5.554; p < 0.001; Fig. 10 and Table 4) [41, 61–64].

## Discussion

Biological similarity to humans is one of the most important characteristics of laboratory animals such as mice and rats. Therefore, these animals are preferred in most experimental studies related to DM. The prevalence of metabolic diseases, mainly DM, has become one of the main concerns related to cardiovascular complications in recent decades. Here, the combined effect of Mel and Exc was investigated on oxidative status, inflammation, apoptosis, and lipid and glucose profile of heart tissue in diabetic rodents [65, 66]. We found that Mel along with Exc increases the level of cardiac SOD, GSH, and GPx enzymes in a mouse model of DM, showing an increase in antioxidant defenses under diabetic conditions. Studies have shown that Exc can lead to an increase in antioxidant enzyme activity in diabetic rats. In addition, Mel can increase the activity of these antioxidant enzymes in STZ-induced diabetic rats. Reduction of free radicals and regulation of antioxidant balance is one of the features of Exc which can eliminate free radicals such as ROS. One of the effective functions of Mel in diabetic conditions is the stabilization of microsomal membranes against oxidative stress [67]. It should be noted that with the activity of Mel, ROS and active nitrogen species (RNS) can be oxidized to N1-acetyl-N2-formyl-5-methoxykynuramine [68]. Mel activates nuclear Nrf2 (NF-E2-related factor 2), which in turn initiates antioxidant mechanisms [68]. In addition, the antioxidant enzyme's key role in redox homeostasis is coherent as the γ-glutamyl tripeptides may serve as a substrate for the GPx/Glutathione reductase (GR)/NADPH system which is directly linked with energy metabolism through the pentose phosphate pathway [69]. As a free radical-producing system, lipid peroxidation is directly related to tissue damage caused by diabetic conditions. Of note, MDA is a suitable factor for the evaluation of lipid peroxidation rate. Studies have reported increased levels of MDA with the progression of diabetic changes. Glutathione provides major protection against oxidative damage by participating in the cellular defense system against oxidative damage. It has been reported that tissue damage caused by various stimuli is associated with glutathione depletion [25, 70].

We also showed that DM increases the expression of inflammatory cytokines such as IL-6, IL-1β, and TNF-α. The use of Mel and Exc reduces these factors and closes them to almost normal levels, indicating the reduction of inflammation changes inside the heart tissue. Studies have shown that TNF-α participates in insulin resistance and ROS production through the regulation of glucolipotoxicity pathways [71, 72]. By neutralizing ROS and RNS, Mel can prevent tissue damage, block transcription factors of pro-inflammatory cytokines, and reduce free radical damage to biomolecules [9]. Myocardial inflammation is also involved in the pathophysiology of diabetic cardiomyopathy [9, 73]. It was suggested that inflammation is the main pathogenic feature and is associated with hyperlipidemia and hyperglycemia [9]. Within the cardiac tissue, inflammatory signaling is usually initiated in response to myocardial injury, because of the overproduction of mitochondrial ROS [9]. Nuclear factor-κB (NF-κB) is a key regulator of inflammatory responses, regulating the expression of pro-inflammatory cytokines in the heart [74]. Pro-inflammatory cytokines are directly responsible for the complications of diabetes and heart disease [74]. Studies have shown that treatment of diabetic mice with Mel led to a significant decrease in the levels of TNF-a, IL-1β, and IL-6 [74–77]. Under diabetic conditions, endothelial function is impaired due to the elevation of TNF-α or IL-6, suggesting that these cytokines can also promote endothelial dysfunction in coronary arteries [78]. Exc can significantly reduce TNF-α and IL-6 levels in diabetic rats [78]. Since Exc significantly affects cellular homeostasis, the levels of cytokines decrease after adaptation to regular exercise [44].

A significant increase of pro-apoptotic proteins such as Bax, Caspase-3, and p53 with a decrease of anti-apoptotic protein Bcl-2 has been observed in the heart tissue of diabetic rats [79]. Data indicated that treatment of diabetic mice with Mel can restore the balance between apoptosis regulatory proteins [79]. Hyperglycemia leads to excessive production and accumulation of ROS in mitochondria, which triggers intrinsic apoptotic signals [80]. It seems that these conditions promote mitochondrial dysfunction in endothelial cells in an AMPK-dependent manner [81]. Accumulation of systemic glucose and byproducts as well as ROS contribute to mitochondrial apoptotic death through the cytochrome C leakage into the cytosol and activation of Caspase-3 [82]. Mel has the potential to reduce mitochondrial dysfunction by the regulation of the AMPK signaling cascade [63]. The reduction of cardiomyocyte damage is associated with the reduction of mitochondrial oxidant stress and apoptosis [63]. The reduction of Caspase-3 by Mel and Exc blunts the deleterious effects of hyperglycemic conditions on cardiac tissue [17].

Exc increases the activity of antioxidant enzymes and cell resistance to oxidative stress [83]. As a correlate, Exc can neutralize oxidative damage, improve insulin sensitivity, and increase glucose metabolism [84]. Also, Exc before ischemia leads to the reduction of pro-apoptotic/anti-apoptotic proteins and inactivation of the Caspase pathway, especially Caspase-3 [85]. One of the important effects of Exc is related to the expression of protein kinase B. These mechanisms can protect the host cells against apoptosis by the phosphorylation of the Bcl-2 family and regulation of pro-apoptotic proteins such as Bax [86, 87].

Mel treatment significantly can reduce hyperglycemia and block hemoglobin glycosylation in diabetic rats [88–90]. Due to insulinogenic and antioxidant activities, Mel can stimulate insulin secretion, regenerate β-cells, or even protects remaining β-cells [91]. One of the therapeutic effects of Mel in diabetic conditions is associated with the reduction of oxidative stress induced by homocysteine [92]. It has been indicated that the elevation of homocysteine accelerates insulin-receptor cleavage and diminishes insulin-resistant conditions [72].

There is a positive correlation between diabetic hyperlipidemia and the occurrence of cardiovascular diseases [93]. Mel has been shown to exert anti-dyslipidemic effects under diabetic conditions [93, 94]. A significant increase in serum triglyceride, TC, LDL-C, and VLDL-C levels along with a decrease in HDL-C levels occurs in DM [95–97]. The underlying mechanism of the Mel cholesterol-lowering effect may be through decreasing cholesterol absorption from the gut or increasing endogenous cholesterol clearance [98]. Mel effectively prevents hyperlipidemia by increasing insulin secretion and lipid storage in fat cells [9]. The accumulation of excess fat in fat cells causes insulin resistance. Under such conditions, the secretion of insulin and adipose cytokines leads to the death of pancreatic beta cells because of free fatty acids [99]. It was suggested that exercise training reduces fat accumulation. Besides, Exc changes the amount of some adipokines and reduces the accumulation of fatty acids, and increases insulin sensitivity [99].

## Conclusions

The occurrence of diabetic conditions is associated with cardiovascular pathologies, especially in cardiomyocytes with the promotion of apoptotic changes, inflammatory responses, oxidative status, etc. These features can affect the normal physiology of the heart, leading to micro- and macro-vascular injuries. Here, systematic review and meta-analysis indicated that co-administration of Mel and Exc can blunt the detrimental effect of DM via the regulation of anti-oxidant capacity, lipid metabolism, inflammatory response, and apoptotic changes, leading to the reduction of cardiomyopathy in diabetic patients.

## Data Availability

All data generated or analyzed during this study and supporting our findings are included and can be found in the manuscript. The raw data can be provided by the corresponding author upon reasonable request.

## Abbreviations

AMPK: AMP-activated protein kinase
CI: Confidence interval
COX-2: Cyclooxygenase-2
DM: Diabetes mellitus
Exc: Exercise
GPx: Glutathione peroxidase
LDL: Low-density lipoprotein
Mel: Melatonin
RNS: Reactive nitrogen species
ROS: Reactive oxygen species
SMD: Standardized mean difference
SOD: Superoxide dismutase
T2DM: T1DM, type 2 DM
T1DM: Type 1 DM
VLDL: Very-low-density lipoprotein
